# Identifying a type of toxic effectors exported by the type VII secretion system to enhance competitive fitness in *Streptococcus suis*

**DOI:** 10.3389/fcimb.2025.1685307

**Published:** 2025-10-14

**Authors:** Qiankun Bai, Jianan Liu, Jie Zhao, Xinming Pan, Yue Zhang, Zongfu Wu, Jiale Ma

**Affiliations:** ^1^ MOE Joint International Research Laboratory of Animal Health and Food Safety, College of Veterinary Medicine, Nanjing Agricultural University, Nanjing, China; ^2^ Key Lab of Animal Bacteriology, Ministry of Agriculture, Nanjing, China; ^3^ World Organization for Animal Health Reference Lab for Swine Streptococcosis, Bacterial Pathogenesis Research Group, Nanjing, China; ^4^ Nanjing Dr. Vet Health Management Co., Ltd., Nanjing, China; ^5^ College of Veterinary Medicine, Henan Agricultural University, Zhengzhou, China

**Keywords:** *Streptococcus suis*, toxin, ESSC, MSE-ExT, T7SSb, competitive fitness

## Abstract

**Background:**

*Streptococcus suis* poses a significant threat to both pig farming and public health, causing severe disease such as septicemia and meningitis. The type VII secretion system (T7SS) delivers toxic effectors to play a crucial role in interbacterial competition and is vital for the zoonotic pathogen *S. suis* to colonize host tonsils effectively.

**Results:**

Here, we identified a type of hypothetical T7SS effector in *S. suis*, which appears to be fragmented toxins lacking the N-terminal YeeF domain, redesignated as MSE-ExTs. MSE (marker for searching effectors) is a conserved sequence at the N-termini of modular effectors showing a diverse range of toxicities targeting NAD +. Cognate WXG100-like and full-length EssC proteins contribute to activate the T7SS secretion. While most MSE-ExTs (MSE-fusing exported toxins) are encoded downstream of a truncated essC and lack cognate WXG100-like genes, they are nonetheless exported and function in interbacterial antagonism, thereby conferring a competitive advantage against bacterial isolates derived from tonsil microbiota. Deletion of the truncated essC could not diminish the MSE-ExT1 delivery, while the full-length EssC1 encoded in T7SS core locus was required for the lethality of MSE-ExT1 to sensitive bacterial cells. MapC2, an upstream small helical protein, shares a nearly identical C-terminal 50-amino acid (aa) sequence with EIC-CR (C-terminal conserved region of effector-paired immunity protein). This conserved fragment harbors a “YxxxD” targeting signal and interacts with the D1 ATPase domain of the non-neighboring EssC, thereby activating the secretion of MSE-ExTs.

**Conclusions:**

This alternative strategy facilitates effectors’ delivery, even for fragmented substrates, highlighting its importance in ensuring the functionality of T7SS.

## Introduction

*Streptococcus suis*, an important zoonotic pathogen (Tan et al., 2019), predominantly affects swine by colonizing the respiratory tract, with a strong presence in the tonsils and nasal passages (Segura, 2020), and even different serotypes of *S. suis* can be found in the same tonsil (Vötsch et al., 2018). In pigs, its infection can lead to arthritis, meningitis, septicemia, and even sudden death (Gottschalk et al., 2010; Goyette-Desjardins et al., 2014). In humans, exposure to infected pigs or contaminated pork products may develop into serious cases such as streptococcal toxic shock-like syndrome (STSLS) (Feng et al., 2010) or bacterial meningitis. To achieve the most optimized survival during infection, *S. suis* exports diverse proteins via secretion systems, such as the general secretory pathway (Sec) and the type VII secretion system (T7SS), to defend against host microbiota antagonism and to interact with host cells (Abdallah et al., 2007; Green and Mecsas, 2016).

T7SS was initially described as an ESX system in the actinobacterial pathogens *Mycobacterium tuberculosis* and *Mycobacterium bovis* (Pym et al., 2003; Stanley et al., 2003; Hsu et al., 2003), but the advancements in next-generation sequencing have facilitated its identification in numerous Actinobacteria (T7SSa) and Firmicutes (T7SSb) bacteria (Mj, 2002). In *S. suis*, a zoonotic pathogen, its T7SSb locus (Lai et al., 2017) has been reported to be critical for colonizing host tonsils effectively (Liang et al., 2022), while the underlying mechanism remains poorly understood. The functions of a given T7SS in interbacterial competition or virulence are determined by the library of its secreted effectors. The canonical T7SS substrates are proteins belonging to the WXG100 family, comprising small helical hairpins that form homo- or heterodimers (Renshaw et al., 2005; Sundaramoorthy et al., 2008). In ESX systems, at least 22 WXG100 proteins have been identified as effectors with diverse pathogenic roles (Mj, 2002; Abdallah et al., 2007; van der Wel et al., 2007; Tak et al., 2021). Among T7SSb-containing bacteria, the *ess* loci of *Staphylococcus aureus*, *Streptococcus intermedius*, *Streptococcus agalactiae*, and *Bacillus subtilis* are extensively characterized (Whitney et al., 2017; Spencer et al., 2021; Klein et al., 2022; Yang et al., 2023; Klein et al., 2024). In these bacteria, WXG100-like proteins often serve as stabilizing and/or targeting factors for LXG proteins, which are larger T7SS substrates. *S. intermedius* and *B. subtilis* produce a variety of LXG proteins fused with diverse C-terminal toxins that have been verified as T7SSb effectors (Whitney et al., 2017; Kobayashi, 2021). More recently, the YeeF domain, also considered as a divergent LXG-like domain (Yang et al., 2023), was identified to constitute a class of T7SSb effectors by fusing with diverse C-terminal toxins (Kaundal et al., 2020; Bowran and Palmer, 2021), including the well-characterized EsaD effector in *S. aureus* (Cao et al., 2016). In particular, TslA, a new type of effector with a reverse domain arrangement (Garrett et al., 2023), was identified in *Staphylococcus*, *Enterococcus*, and *Listeria*. Despite the identification of many T7SS effectors in different bacterial species, their biological activities are still not sufficient to fully explain the divergent functions of T7SSs, suggesting that there are many unknown effectors awaiting to be studied.

Numerous studies have uncovered the roles of T7SSb in mediating cell damage against neighboring prokaryotes (Whitney et al., 2017) via a contact-dependent mechanism of delivering toxic effectors, including nucleases that cleave DNA or RNA (Cao et al., 2016), pore-forming toxins and phospholipases that target cell membranes (Spencer et al., 2021), and NADases (Sun et al., 2015) that affect cellular energy balance or interrupt cell division. These effectors display a complex diversity in terms of domains that exhibit toxin activity (Zhang et al., 2011; Ting et al., 2018), yet they possess a common architecture with conserved N-terminal trafficking domains that are fused with polymorphic toxin domains at the C-termini (Zhang et al., 2012; Jamet and Nassif, 2015; Klein et al., 2020). Conserved domains such as WXG100, WXG100-like SACOL2603, and LXG (Bowran and Palmer, 2021; Dedrick et al., 2021; Klein et al., 2022) can serve as markers to identify the potential T7SS effectors throughout the bacterial genomes. The C-termini and the downstream regions of *essC* genes are frequently subject to genetic rearrangement, which results in the formation of a large number of WXG100 and LXG variants, serving as a potential effector reservoir in multiple species (Spencer et al., 2021; Bowman and Palmer, 2021; Bowran and Palmer, 2021). Various EssC variants are known to specifically recognize different suites of secreted effectors (Warne et al., 2016; Jäger et al., 2018; Bowran and Palmer, 2021), while numerous predicted effectors do not appear alongside a full-length cognate EssC for the activation of a T7SS apparatus. Further studies are necessary to determine whether the biological functions of these truncated EssC and effectors are retained.

The T7SSb apparatus is mainly composed of six core components, namely, EsxA, EsaA, EssA, EsaB, EssB, and EssC (Spencer and Doran, 2022), and has been reported to export effectors via two substrate recruitment strategies. In a subset of *S. aureus* strains, the substrates EsxB, EsxC, and EsxD recruit the co-occurred downstream EsaD effector, subsequently activating the T7SSb secretory channel via a “YxxxE/D” secretion signal (Mietrach et al., 2020; Yang et al., 2023). Analogously, *S. intermedius* deploys DUF3130 and DUF3958 proteins activating the T7SSb machinery by an “FxxxD” signal peptide, thereby facilitating the secretion of downstream cognate LXG effectors (Klein et al., 2022). In *B. subtilis*, the secreted core component EsxA and the N-terminal LXG domain of the antibacterial toxin YxiD interact with the EssB pseudokinase domain, which in turn interacts with the FHA domains of the EssC ATPase protein (Tassinari et al., 2022). D2 and D3 ATPase activities are required for the secretion of LXG effectors in T7SSb of *S. aureus* (Mietrach et al., 2020; Yang et al., 2023) and *S. intermedius* (Klein et al., 2022), but they are dispensable for LXG secretion in the Yuk system of *B. subtilis* (Ramsdell et al., 2015).

In this study, we identified a novel type of putative effector in *S. suis*, which appears to originate from toxin fragments lacking the N-terminal YeeF domain, redesignated as MSE-ExTs. Most of these MSE-ExTs are predominantly located downstream of truncated *essC* genes within the T7SSb loci and demonstrate export capability to facilitate interbacterial competition in *S. suis*. Our subsequent works aimed to clarify which pathways were employed by these effectors to overcome the challenges posed by the absence of a full-length cognate EssC variant. The conserved EIC-CR (DUF6572) domain, located at the C-termini of paired immunity proteins, was verified to be effective in ensuring the successful delivery of MSE-ExTs via binding to the D1 ATPase domain of the non-neighboring EssC encoded in T7SS core locus. This alternative strategy within effector–immunity pairs significantly broadens our comprehension of the T7SSb secretion mechanism, thereby enhancing the repertoire of T7SSb effectors.

## Methods

### Bacterial strains and growth conditions

The *S. suis* isolates K56-WJ, K11-WJ, and 128-2–1 were stored in the WOAH Reference Laboratory for Swine Streptococcosis. All these strains grew at 37 °C, 5% CO_2_ in Todd–Hewitt broth (THB, Oxoid Cheshire, UK) or THB agar (THA). When required, antibiotics and chemicals were added at the following concentrations: spectinomycin (Sigma-Aldrich) at 100 μg/mL, chloramphenicol (Sigma-Aldrich) at 5 μg/mL, and sucrose at 10% (wt/vol). A detailed list of bacterial strains and plasmids used in this study can be found in **Supplementary Table S1**.

### DNA manipulations and plasmid construction

All oligonucleotide primers synthesized by Genscript Biotechnology Co., Ltd. (Nanjing, China) are listed in **Supplementary Table S2**. DNA amplification, ligation, and electroporation were performed as previously described (Ma et al., 2017), unless otherwise indicated. Deletion mutants were constructed using the natural transformation method, according to the previous studies of our lab (Zhu et al., 2019). The complemented strains were constructed with the plasmid pSET2 vector. The target gene fused with the T7SSb promoter was cloned into the pSET2 vector using the ClonExpress Ultra One-Step Cloning Kit (Vazyme). For gene expression, the targeted ORF was cloned into the pBAD-HisA plasmid, and the *Escherichia coli* Top10 was used to further express the target gene.

### Growth curves for the bacterial toxicity assay

The vector pBAD/HisA (Invitrogen) was used to carry the indicated genes for effectors’ expression and effector–immunity pairs’ co-expression (Ma et al., 2017; Gu et al., 2024). For *E. coli* growth curve detection, Top10 cells harboring the indicated recombinant plasmid were grown overnight and sub-inoculated to a starting OD_600_ value of 0.05 in LB at 37 °C with shaking for 2.5 h. The target gene was then induced with 0.2% (w/v) L-arabinose and the vector pBAD/HisA served as a control. Cell growth was tracked by measuring the OD_600_ value every 2 h.

### Western blot assays identify the protein secretion of the MSE-ExT1 effector

The secreted proteins of each *S. suis* strain were extracted as described previously (Kneuper et al., 2014). Bacterial culture (500 mL) was incubated to the logarithmic phase (OD_600_ value at 1.0), then centrifuged at 12,000 ×*g* for 10 min. Supernatant samples were further extracted using the classical TCA-acetone approach to acquire the secreted proteins. The above extracted samples were analyzed by conventional Western blot assays using the anti-ExT1 antibody to identify the secretion of JNE31_RS11280/MSE-ExT1. A SLY-specific antibody was used, and a cross-reactive band was shown as a loading control (Zhang and Lu, 2007). The anti-ExT1 serum was prepared by Zoonbio Biotechnology Co., Ltd. using the mice immunized with the nontoxic RS11280_G477V_ protein.

### Determination of intracellular NAD^+^ levels

*E. coli* Top10 cells harboring the indicated recombinant plasmids were cultured in LB medium at 37 °C to mid-log phase (OD_600_ value at 0.8) and then were induced with 0.2% (w/v) L-arabinose for protein expression. After 1 h of incubation, cultures were diluted to an OD_600_ value at 0.6, and the bacterial cells of 3 mL dilution were harvested by microcentrifugation. The intracellular NAD^+^ levels were measured using the NAD/NADH Assay Kit (Colorimetric, ab65348) per the manufacturer’s instructions (Abcam). The relative NAD^+^ concentrations were calculated by comparing to a vector control strain (Top10 cells carried with pBAD-HisA).

### Interbacterial competition assay

For contact-dependent interbacterial antagonism (Whitney et al., 2017), the donor (such as K56-WJ, K11-WJ, 128-2-1, or their mutant strains) and recipient (such as Δ*mse-ext1&ei1*, Δ*mse-ext1*, Δ*mse-ext7&ei7*, Δ*mse-ext7*, Δ*mse-ext12&ei12*, or Δ*mse-ext12*) strains were diluted in THB to a starting OD_600_ value at 0.6, then mixed at a volume ratio of 10:1. The supernatant was discarded by centrifugation at 7,000 ×*g* and resuspended in 100 μL of phosphate-buffered saline (PBS). Then, 8 μL of the mixture was spotted on the LB plate and incubated at 37 °C and 5% CO_2_ for 22 h. Finally, the spotted cells were re-suspended and serially diluted in PBS, and cultured on THB plates to select recipient colonies using the corresponding antibiotic. The colony forming unit (cfu) of the survival recipient cells was counted on chloramphenicol plates.

### Bacterial two-hybrid analysis

Bacterial two-hybrid assays were performed using the BACTH (Bacterial Adenylate Cyclase Two-Hybrid) System kit (Euromedex, France) according to the supplier’s instructions. The recombinant plasmids of pKT25 and pUT18C listed in **Supplementary Table S1** were introduced into *E. coli* BTH101 for subsequent analysis. The interaction was visually monitored by the appearance of a blue color due to the activity of b-galactosidase on X-gal (5-bromo-4-chloro-3-indolyl-b-D-galactopyranoside; 20 mg/mL). After 36 h, the products were transferred into 96-well plates to display the results. The bacterial cells (*E. coli* strain BTH101) transformed with plasmids pKT25-zip and pUT18C-zip served as positive controls, and those with pKT25 and pUT18C served as negative controls.

### Bioinformatics analysis

The bioinformatics verification of retrieved MSE or EIC-CR proteins (Pei et al., 2008; Camacho et al., 2009) was performed using the UniProtKB release 2024_01 (https://www.uniprot.org/) as previously described (UniProt Consortium, 2019). Their functional prediction was performed using SWISS-MODEL (Waterhouse et al., 2018) (https://www.swissmodel.expasy.org/, probability >50%) or Phyre2 (Powell et al., 2025) (confidence >95%). Monomeric 3D protein structure predictions were performed by AlphaFold v3.0.0 running on our local server with default parameters (Jumper et al., 2021). The phylogenetic tree was constructed using MEGA 7.0 with the neighbor-joining method (1,000 bootstrap replicates; Poisson correction) and visualized by iTOL (Letunic and Bork, 2021) (https://itol.embl.de).

### Molecular docking

To investigate the interacting patterns between ligands (EIC-CR/MapC2) and the D1–3 ATPase encoding region of EssC, Cluspro 2.0 (Kozakov et al., 2017), AlphaFold3, and Autodock Vina (Trott and Olson, 2010) were used for semi-flexible docking to screen the orientation pattern of ligands in the binding putative pocket of the D1/2/3 domain and identify the residues involved in the interaction, and all ligands adopted the same parameters for docking. Outputting the top six conformations for each ligand, the most reliable binding poses were selected according to the interaction energy. All results were analyzed and visualized using PyMOL v1.8 (Schrödinger, 2015). A similar strategy was applied to investigate the interacting patterns between MSE and EsaA, and between MSE and MapC2.

### RluC (Renilla luciferase) translocation assay

C-terminal translational fusions of RluC with each designated fragment (such as EIC-CR-MSE and MapC2-MSE) were constructed by using pKSM410-*rluC* (operon controlled by tetracycline). The indicated *S. suis* strains and an isogenic T7SSb-negative mutant (Δ*esaA*) harboring the RluC fusion plasmids were grown for 2 h in THB medium, and then anhydrotetracycline was added for another 2-h incubation to induce fusion proteins’ production and secretion. Subsequently, the culture supernatant was concentrated more than 50-fold by ultrafiltration (Millipore, 10 kDa cutoff) and then was loaded with the fluorescent substrate coelenterazine for 1-h incubation. Using the Renilla luciferase reporter gene assay kit (Beyotime), substrate oxidation catalyzed by RluC was detected by a luminometer (M200Pro, Tecan, Switzerland) at an integration time of 1,000 ms, indicating translocation of the effector fusion protein.

### Pull-down assay

A pull-down assay was used to confirm the direct interaction of the indicated protein pair. Taking EIC-CR and D1_EssC_, for example, 1.5 μM of pure rEIC-CR-GST protein was incubated in 600 μL of binding buffer (50 mM HEPES, pH 7.5, and 250 mM NaCl) with 0.75 μM rD1_EssC1_-His for 4 h with rotation and 0.1 mM PMSF added every hour. Anti-GST-Tag antibody (Abmart M20025) was diluted in 600 μL of binding buffer at 1:500 and incubated for 2 h on a rotator with prewashed Protein A/G Magnetic Beads (MedChemExpress). The beads were then washed three times. The protein mixtures were incubated with the beads coated with the Anti-GST-Tag Antibody for 3 h, and then the beads were washed 6 times with PBST. Bound proteins were eluted with 5 × SDS sample loading buffer, boiled for 10 min, and loaded onto a 12% SDS-PAGE gel for Western blot analysis. A similar strategy was applied to determine the interactions of another protein pair.

### SPR analysis

Surface plasmon resonance (SPR) experiments were performed using a Biacore T200 instrument (GE Healthcare) at 25 °C as described previously (Wojtowicz et al., 2020). rD1, rD2, rMSE-C, and rMSE-N were immobilized on the CM5 sensory chips at 600 response units (RUs), respectively. An uncoated “blank” channel was used as a negative control. The EIC-CR/MapC2 analyte was diluted in running buffer [10 mM HEPES, pH 7.4, 150 mM NaCl, 3 mM EDTA, 0.05% Tween 20, and 5% dimethyl sulfoxide (DMSO)] and injected at different concentrations (twofold dilutions; 4–256 nM) at a flow rate of 30 µL min^−1^. The resulting data were analyzed using Biacore T200 evaluation software (GE Healthcare).

### Statistical analysis

The data were analyzed using Prism 8.0 (GraphPad). A two-tailed Student’s *t*-test was used for interbacterial competition and recipient survival assays. *P* values of less than 0.05 were defined as significant and indicated with one or more asterisks (*P < 0.05, **P < 0.01, ***P < 0.001).

## Results

### Identification of a toxic effector mediating interbacterial antagonism in *S. suis*

**Figure 1:**
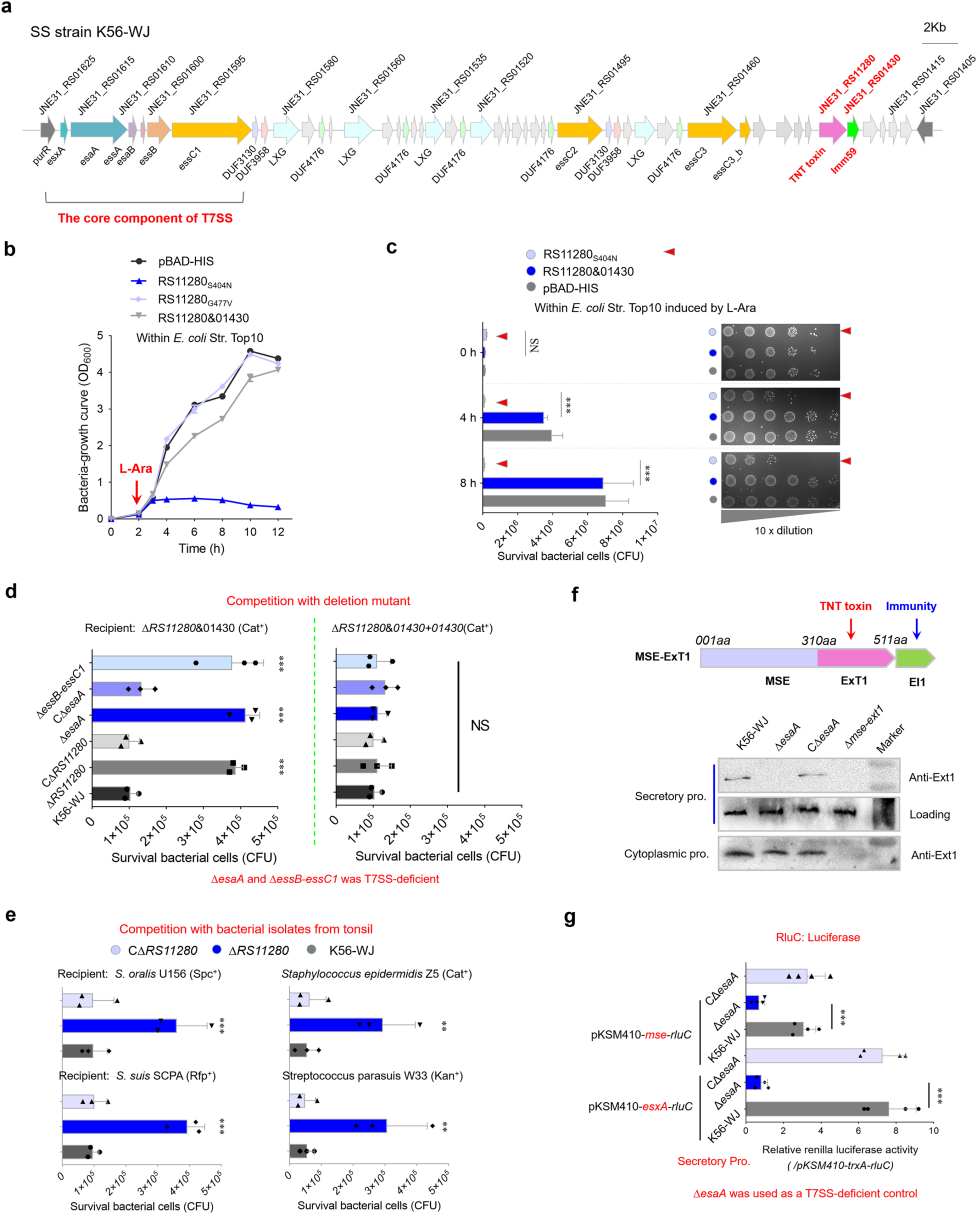
Identification of a novel antibacterial effector secreted by T7SSb. **(a)** Graphical depiction of the T7SSb gene locus in *S. suis* strain K56-WJ. The direction of the arrows indicates the direction of transcription. Genes encoding the conserved EssC domain are represented by orange. **(b)** Growth curves of *E. coli* cells expressing the indicated proteins. The cultures were induced by L-arabinose at the indicated time (shown by the red arrow). **(c)** Monitor the bacteriocidal or bacteriostatic activity of JNE31_RS11280. The *E. coli* cells harboring the indicated plasmids were incubated with 0.2% L-arabinose (inducing conditions). Cell growth was tracked by measuring the survival bacterial cells (CFU) every 2 h **(d)** Interbacterial competition assays between indicated *S. suis* donor and recipient strains. The donor and recipient were mixed at 10:1 and incubated on an LB culture plate for 22 h Owing to its high toxicity, cloning JNE31_RS11280 into a pBAD-His vector under an arabinose-inducible promoter was unsuccessful, but a recovery (with reduced toxicity) was achieved by introducing an S404N mutation. Another nontoxic mutation, G477V (spontaneously introduced into a recovery during the constant repetition of *JNE31_RS11280* cloning), completely abrogating the toxicity of JNE31_RS11280, was thus used as a negative control. **(e)** Interbacterial competition with bacterial strains isolated from the tonsil of healthy pigs. These ExT1-sensitive isolates were screened from the microbiota of pig tonsil. The assays were performed as outlined in the procedure of **Figure 1d**. **(f)** Secretion identification of MSE-ExT1 using Western blot analysis in the indicated strains. The anti-ExT1 serum was prepared by Zoonbio Biotechnology Co., Ltd. using the mice immunized with the nontoxic RS11280_G477V_ protein. An SLY-specific antibody was used as loading control for secretion proteins in *S. suis*. Since there were three *essC* variants in the T7SS locus, the mutant Δ*esaA* was employed as a T7SS-deficient control. Similar results have been verified in two other independent experiments. **(g)** RluC translocation assays of T7SSb MSE effectors in *S. suis* strains. The concentrated culture supernatants of indicated strains were measured by using the Renilla Luciferase Reporter Gene Assay Kit (Beyotime). TrxA, a cytoplasmic protein, was used as a control of background fluorescence value (**Supplementary Figure S3**). The fluorescence values of cytoplasmic samples showed a similar level in different strains (**Supplementary Figure S3**). CΔ*esaA* is a complementary strain of Δ*esaA*. **(b–e, g)** Statistical significance was calculated using a two-tailed Student’s *t*-test. Error bars represent the mean ± SD of three biological repeats. **P < 0.01; ***P < 0.001; NS, not significant.

The bactericidal activity of T7SSb is attributed to the delivery of toxic effectors, which are paired with cognate immunity proteins encoded downstream to prevent self-intoxication (Whitney et al., 2017; Kobayashi, 2021). In *S. suis* strain K56-WJ, an Imm59 family immunity protein (JNE31_RS01430) was found in the T7SSb locus (**Figure 1a**); thus, its upstream protein (JNE31_RS11280) was predicted to be a potential effector. Because of its high toxicity, cloning JNE31_RS11280 into a pBAD-His vector was unsuccessful, but a successful recovery was accomplished by introducing an S404N mutation, which occurred spontaneously during the repeated attempts to clone JNE31 RS11280. Another nontoxic mutation, G477V, was also screened out using the above method and was used as a negative control in a succeeding study. AlphaFold-predicted structures of these two mutations showed coincident superimpositions with the JNE31 RS11280 (**Supplementary Figure S1**), suggesting that the reduction in toxicity may be caused by other reasons. The expression of JNE31_RS11280_S404N_ significantly inhibited the growth of the *E. coli* Top10, but this deficiency was restored by co-expression with the Imm59 protein, indicating that these two proteins form an effector–immunity pair (**Figures 1b, c**). To verify the role of this effector in mediating interbacterial antagonism, a recipient strain without any growth deficiency (**Supplementary Figure S2**) was generated by the deletion of individual *JNE31_RS11280&01430* gene pairs. Unlike the wild-type strain, Δ*RS11280* significantly impaired its capacity to kill the recipient strain Δ*RS11280*&*01430* on the plate, and its complementation fully restored this deficiency (**Figure 1d**, left panel). This killing capacity was not shown in the assays using the recipient strain Δ*RS11280*&*01430 + 01430*, indicating that the complementary *RS01430* neutralizes the toxicity of RS11280 (**Figure 1d**, right panel). Similar results were also observed using several ExT1-sensitive isolates from the microbiota of pig tonsil as recipient strains (**Figure 1e**), suggesting that *JNE31_RS11280* may facilitate *S. suis* to obtain a competitive advantage over the specific members of the tonsil microbiota.

Depletion of EsaA or EssB and EssC1 by gene deletion impaired the killing of recipient strain mediated by JNE31_RS11280 (**Figure 1d**), while EsaA complementation could restore this deficiency of Δ*esaA* to a similar level with the wild-type strain, determining that this putative effector is secreted via the T7SSb pathway. Notably, the RS11280-N [1–310 amino acids (aa)] sequence is commonly present in *S. suis* genomes, implying its potential utility as a marker for searching effectors (MSE); thus, we redesignated the JNE31_RS11280&01430 pair as MSE-ExT1 (MSE fused with exported toxin 1) and EI1. Western blot analyses of the culture supernatants using the anti-ExT1 antibody confirmed the export of MSE-ExT1 in both wild-type and CΔ*esaA* strains, while no export was observed in Δ*esaA* and Δ*mse-ext1* strains (**Figure 1f**). These differences were not observed among wild-type, Δ*esaA*, and CΔ*esaA* strains in the cytoplasmic samples. Since there were three *essC* variants in the T7SSb locus, the mutant Δ*esaA* was employed as a T7SSb-deficient control here. EsxA, a hallmark of functional T7SSb, was tagged with RluC luciferase using a luciferase reporter plasmid pKSM410*-esxA-rluC*, and its secretion characteristics in the indicated strains were observed by measuring the fluorescence of culture supernatants. The plasmid pKSM410-*trxA-rluC* was used as a negative control to demonstrate the background fluorescence value (**Supplementary Figure S3**). As shown in **Figure 1g**, the deletion of *esaA* caused a significant decrease in fluorescence from EsxA-RluC within the cultural supernatant, while complementation could restore this deficiency to a similar level of the wild-type strain, suggesting that this luciferase reporter system could be used to measure the secretion of effector MSE-ExT1. Fluorescence signals from MSE-RluC could be measured in the culture supernatants of both wild-type and CΔ*esaA* strains, with values surpassing those of Δ*esaA*, albeit lower than those of EsxA-RluC (**Figure 1g**). All these findings suggest that MSE-ExT1 is a substrate secreted in a T7SSb-dependent manner.

### MSE-ExTs harboring diverse NADase toxins are widely prevalent in *S. suis*

**Figure 2:**
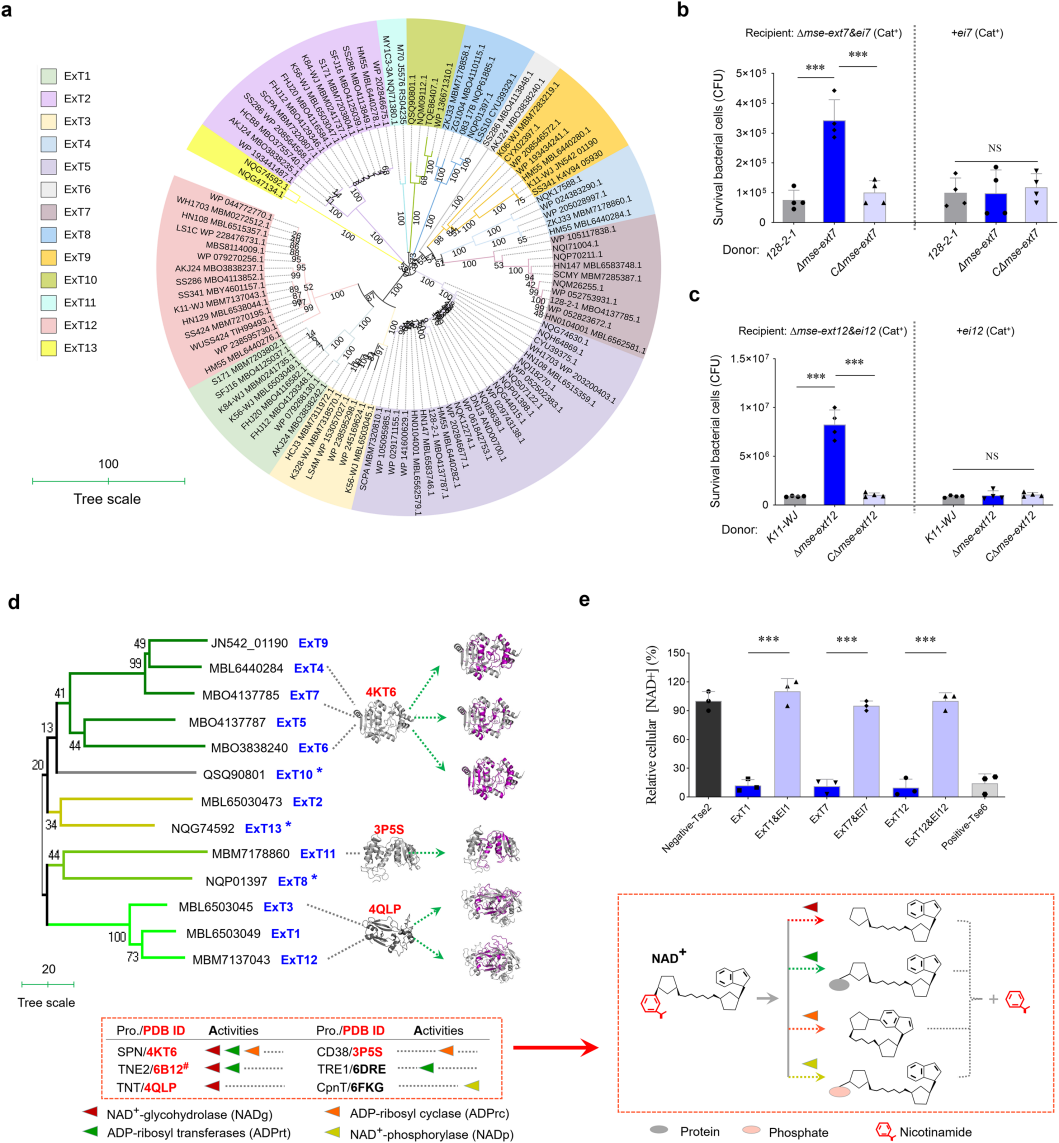
Identifying more MSE-ExT effectors across the *S. suis*. **(a)** Phylogenetic analysis of 104 randomly selected MSE-ExT effectors wildly encoded in *S. suis* isolates. A neighbor-joining tree was constructed based on the C-terminal extension of MSE-ExT effectors using the MEGA software version 7.0. Detailed information is shown in **Supplementary Figure S4**. **(b, c)** Identification of the antibacterial activities of MSE-ExT7 and -ExT12 using the outcome of interbacterial competition assays between the indicated *S. suis* donor and recipient strains. Details are provided in the legend to **Figure 1d**. **(d)** Identification of MSE-ExT effectors harboring diverse NADase toxins in *S. suis* isolates. Ribbon depiction showing the superimposition of AlphaFold3-predicted ExT (colored purple) and the corresponding best-matching template (colored gray) is presented on the right, and detailed information is shown in **Supplementary Figure S8**. All of the best-matching templates were annotated as NADases at the bottom of the phylogenetic tree, their NAD^+^-consuming activities are classified into four varieties (triangles marked in four different colors) as in previous studies (Roussin and Salcedo, 2021), and their cleavage sites and products were exhibited by diagrams on the right. ^#^The annotated function is only predicted but lacks experimental validation. **(e)** Relative NAD^+^ levels were measured using the NAD/NADH Assay Kit (Colorimetric, ab65348, Abcam) in *E. coli* cytoplasm expressing the indicated proteins. Tse2 and Tse6 were used as the negative and positive controls as in a previous report (Jc et al., 2017), respectively. **(b, c, e)** Error bars represent the mean ± SD of three biological repeats. ***P < 0.001.

A BLASTP retrieval process was performed using the conserved MSE sequence as a template against all *S. suis* genomes from this study and the GenBank database (**Supplementary Table S3**). The initial library consisted of more than 300 target proteins (**Supplementary Table S4**), which fuse with diverse toxic domains at their C-termini. Numerous retrieved proteins encoded by the *S. suis* strains from the same serotype showed almost the same sequence and were, thus, removed from the list for further study. A phylogenetic analysis of 104 selected samples divided their various C-terminal fusions into 13 distinct clades (**Figure 2a** and **Supplementary Figure S4**) designated as ExT1-13, underscoring the extensive prevalence of MSE-ExT effectors in *S. suis* and emphasizing the conserved N-terminal MSE sequence as a novel marker for T7SSb effectors. We further randomly selected four strains for genetic neighborhood analysis and found that a single T7SSb locus may encode multiple MSE duplications with exchangeable ExTs (**Supplementary Figure S5**). Of these, MSE-ExT7 and -ExT12 were randomly selected and confirmed as functional T7SSb toxic effectors to kill the indicated recipient strains experimentally (**Figures 2b, c** and **Supplementary Figure S6**). Their killing capacity were not shown in the assays using the recipient strains Δ*mse-ext7&ei7+ei7* and Δ*mse-ext12&ei1+ei12*, indicating that the immunity proteins EI7 and EI12 neutralize the toxicities of ExT7 and ExT12 (right panels of **Figures 2b, c**), respectively. Interestingly, almost all types of ExTs (except for ExT8, ExT10, and ExT13, which lacked comparable homologs) exhibited sequence identities and structural similarities with the well-known NADase proteins (**Figure 2d**, left panel, and **Supplementary Figure S7**), which cover all groups currently reported to degrade the substrates with different modifications and leave divergent products (**Figure 2d**, right panel) (Roussin and Salcedo, 2021). The NADase activities of ExT1, ExT7, and ExT12 were confirmed by detecting significant reductions in intracellular NAD^+^ levels upon induction of their expression, but not in the control groups co-expressing their cognate immunity proteins (**Figure 2e**). The positive control Tse6, a previously characterized interbacterial NADase toxin (Whitney et al., 2017), was used to mirror the extent of NAD^+^ depletion, while the negative control Tse2, an unrelated bacteriostatic toxin, was unaffected by the intracellular NAD^+^ level. These findings suggested that NADase toxins may be a major family of MSE-ExT effectors in *S. suis*.

### Identification of the small helical proteins related with MSE-ExTs

**Figure 3:**
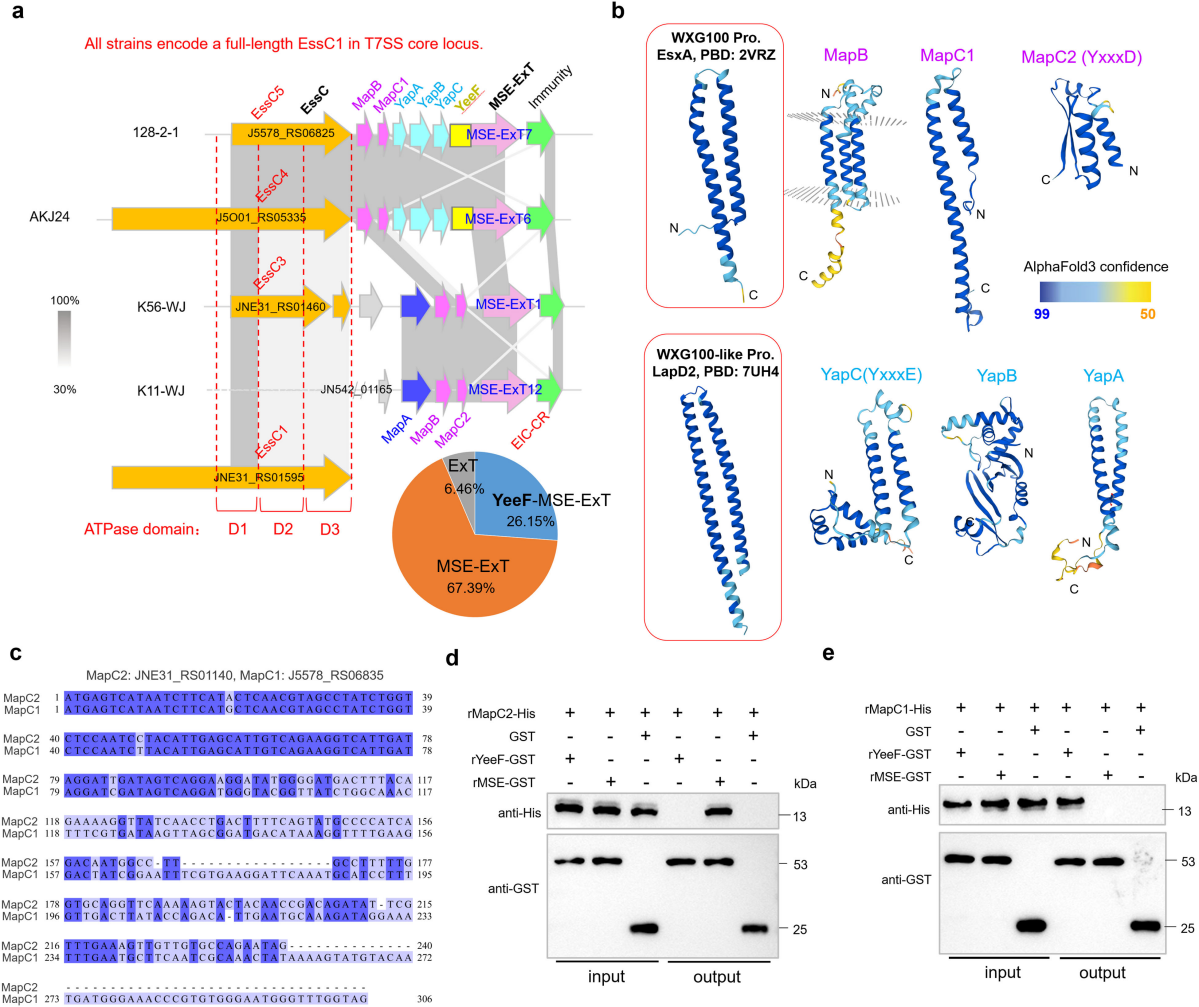
Identification of a conserved adaptor-like domain fused with the C-termini of diverse immunity proteins. **(a)** Genetic neighborhood analyses of *mse*-*ext* loci in several selected *S. suis* strains based on the GenBank annotations. **(b)** A sequence alignment of *mapC1* (J5578_RS06835) and *mapC2* (JNE31_RS01440). **(c)** AlphaFold3-predicted structures of Map (MSE-associated protein) and Yap (YeeF-associated protein) proteins. Thereinto, MapC, YapA, and YapC showed structural similarity with the WXG100-like small helical protein. **(d)** Pull-down assay detected the interaction between MapC2 (JNE31_RS01440/WP_153309225.1) and the YeeF (1–210 aa of YeeF-MSE-ExT7 encoded by strain 128-2-1) or MSE fragment (211–520 aa of YeeF-MSE-ExT7). Purified rMapC2-His was incubated with rYeeF-GST or rMSE-GST, and protein complexes were captured by GST magnetic beads, washed, and eluted in the sample buffer. Empty GST-tag was used as negative control. Fractions were probed with anti-GST and anti-His antibodies. **(e)** Pull-down assay detected the interaction between MapC1 (WP_022540707.1) and the YeeF or MSE fragment.

The genetic neighborhoods of representative *mse-ext* genes in strains HN147, 128-2-1, K11-WJ, and K56-WJ showed that several MSEs fuse with an N-terminal divergent YeeF (LXG-like) domain (Yang et al., 2023) to produce the extended versions, such as the YeeF-MSE-ExT7 and YeeF-MSE-ExT6 (**Figure 3a**), thus suggesting that MSE-ExTs may be fragments of YeeF-related effectors. Further analyses based on the data of **Supplementary Table S4** found that most samples were classified as typical MSE-ExTs (67.39%), and only a few ones belong to YeeF-MSE-ExTs (26.15%). Otherwise, several proteins encoded within the retrieved loci lack both MSE and YeeF domains, while containing the predicted toxin domains, and thus were identified as ExTs (6.46%). It should be noted that most *mse-exts* and *yeeF-mse-exts* are encoded downstream of a truncated *essC* gene. Unexpectedly, the adjacent EssCs of YeeF-MSE-ExTs harbor different C-termini compared with the ones upstream of the typical MSE-ExTs (**Figure 3a**). Furthermore, the loci of *mse-exts* and *yeeF-mse-exts* encode different small helical proteins. Three conserved genes, designated as *mapABC* (encoding MSE-associated protein ABC), are located upstream of the typical *mse-ext* genes, with two of them (*mapB&C*) also present in the loci encoding YeeF-MSE-ExTs (**Figure 3a**). The *mapC* genes from two types of loci share a conserved N-terminal sequence, while showing significant sequence divergences in their C-terminal region (**Figure 3b**), and were thus renamed as *mapC1* and *mapC2*. Otherwise, three additional conserved genes located upstream of *yeeF-mse-exts* were designated as *yapABC* (encoding YeeF-associated protein ABC). YapABC share low sequence identities (~20%) with the small helical proteins of the reported effector EsaD (YeeF toxin) in *S. aureus*, but are significantly different from the DUF3130 and DUF3958 small helical proteins related with LXG effectors in *S. intermedius*. In these neighborhoods, MapC1, YapA (YeeF-associated protein A), and YapC (YxxxE) were identified as WXG100-like proteins based on structure prediction (**Figure 3c**), suggesting their potential roles in facilitating effectors’ secretion via the T7SSb apparatus. MapC2 does not have a typical structure of a WXG100-like protein, but shares sequence identities with the N-terminus of MapC1 and contains a T7SS targeting signal “YxxxD”. Pull-down analysis demonstrated that MapC2 specifically interacted with the typical MSE sequence (211–520 aa of YeeF-MSE-ExT7 encoded by strain 128-2-1), while its interaction with the YeeF domain (1–210 aa of YeeF-MSE-ExT7) was absent (**Figure 3d**). In contrast, the specific interaction of MapC1 was identified with the YeeF domain, but not with the MSE sequence (**Figure 3e**).

### The C-terminal conserved region of immunity proteins plays vital roles for the delivery of MSE-ExTs

**Figure 4:**
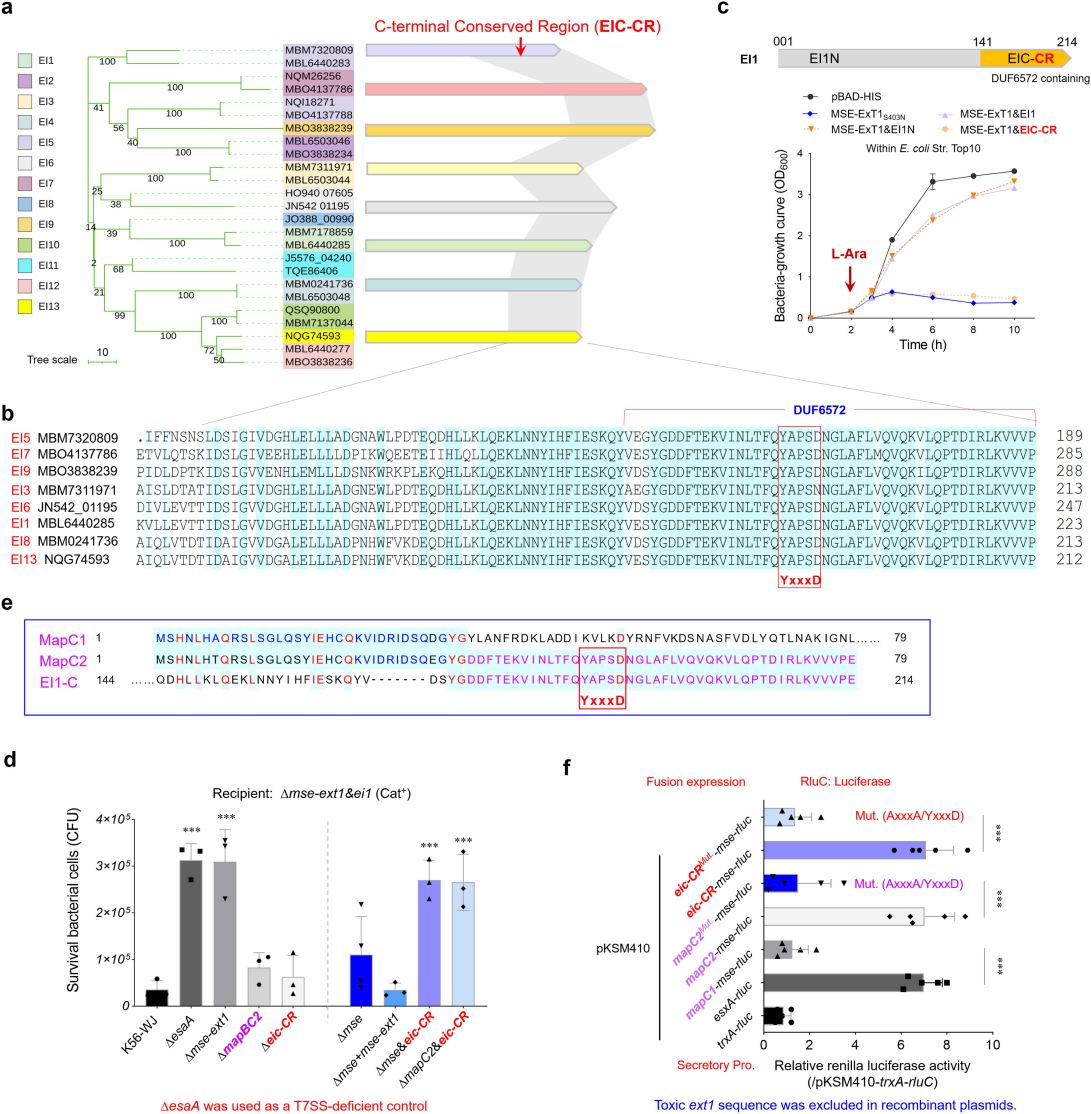
Assessing the roles of EIC-CR and MapC2 in the delivery and toxicity of MSE-ExTs. **(a)** A phylogenetic tree of 25 cognate immunity proteins of the MSE-ExT effectors randomly selected from **Figure 2**a. The right panel shows the sequence identities of immunity proteins from different clades. The gray shadow shows the high sequence identity (>80%) between the two compared fragments. **(b)** A sequence alignment of eight representative EIC-CR was selected according to **Figure 4a**. The mapped residues were shaded sky blue. **(c)** Growth curves of *E. coli* cells expressing the indicated proteins to detect the roles of EIC-CR and EI-N in the neutralization of ExT1 toxicity. **(d)** Effects of MapBC2, EIC-CR, and MSE on interbacterial competition. The assays were performed as outlined in the procedure of **Figure 1d**. **(e)** Amino acid sequence alignment of EI1-C (C-terminal region of EI1), MapC1 (WP_022540707.1), and MapC2 (WP_153309225.1). The mapped residues were shaded sky blue and labeled dark blue, purple, or red. **(f)** RluC translocation assays analyzing the roles of MSE, MapC1, MapC2, and EIC-CR for substrate secretion in *S. suis*. The fluorescence ratio of an indicated reporter plasmid and negative control pKSM410-*trxA-rluC* was calculated in the *mapABC2-mse-ext1-ei1* deletion mutant derived from strain K56-WJ. Mut. (AxxxA/YxxxD) means that “YxxxD” was replaced with “AxxxA”. Details are provided in the legend to **Figure 1g**. **(c, e, f)** Error bars represent the mean ± SD of three biological repeats. **P < 0.01; ***P < 0.001.

A phylogenetic analysis of 25 randomly selected immunity proteins showed that they are divided into 13 clans, consistent with the genetic classification of ExT toxins (**Figure 4a**), supporting the predicted cognate effector–immunity pairs. Most immunity proteins possess a conserved C-terminal region, here designated as EIC-CR. The EIC-CR sequences from different *S. suis* strains share over 85% identities within 100 amino acid sequences (**Figure 4b**) and was recently annotated as a DUF6572 domain. In the immunity protein EI1, the N-terminal EI1N is already sufficient to completely restore the growth deficiency of *E. coli* caused by MSE-ExT1 toxin expression, while EIC-CR/DUF6572 has nothing to do with toxicity neutralization (**Figure 4c**). Therefore, the N-terminal region of EI is responsible for the basic function of immunity proteins in avoiding self-intoxication, whereas the conserved EIC-CR may have a different, yet to be determined, special role in effector–immunity pairs. Startlingly, the deletion of neither *mapBC2* nor *eic-CR* was able to completely abrogate the killing of the recipient strain conferred by the MSE-ExT1 (**Figure 4d**). EIC-CR shares sequence identities with the upstream MapC2, but not MapC1 (**Figure 4e**). The high-sequence identities between the C-terminus of MapC2 and EIC-CR may provide alternative options to facilitate the secretion of effectors. Notably, a conserved “YxxxD/E” motif, recognized as the targeting signal for EssC activation and effector export in diverse WXG100 and WXG100-like proteins (Champion et al., 2006; Daleke et al., 2012), is encoded in EIC-CR and MapC2. All these findings suggest the EIC-CR and MapC2 may play vital roles in the recruiting MSE-ExT effectors.

Although deletion of *eic-CR* sequence alone did not completely abrogate the interbacterial competition using the Δ*mse-ext1*&*ei1* as the recipient (**Figure 4d**), double deletion of *mse*&*eic-CR* or *mapC2*&*eic-CR* completely impaired the competition advantage to the similar levels of strain Δ*mse-ext1*. A series of luciferase reporter plasmids were then introduced into the wild-type strain K56-WJ, and their secretory protein samples were prepared. The fluorescence ratio of an indicated reporter plasmid and negative control pKSM410-*trxA-rluC* was calculated. As shown in **Figure 4f**, the inclusion of either *mapC2* or *eic-CR* in the pKSM410**-** *mse* **-** *rluC* construct significantly enhanced the relative values to a level comparable to that of pKSM410**-** *esxA* **-** *rluC*. However, the fusion protein MapC1-MSE-RluC exhibited a similar value with the negative control TrxA-RluC (~1.0). Compared with the EIC-CR-MSE-RluC fusion protein, the detected value of EIC-CR^Mut. (AxxxA/YxxxD)^-MSE-RluC was significantly decreased to a level similar to that of MapC1-MSE-RluC (**Figure 4f**), and similar results were also observed between MapC2-MSE-RluC and MapC2^Mut. (AxxxA/YxxxD)^-MSE-RluC, indicating that the exportation of fusion proteins have been completely abrogated.

### EIC-CR/DUF6572 specifically interacts with the D1 ATPase domain of EssC

**Figure 5:**
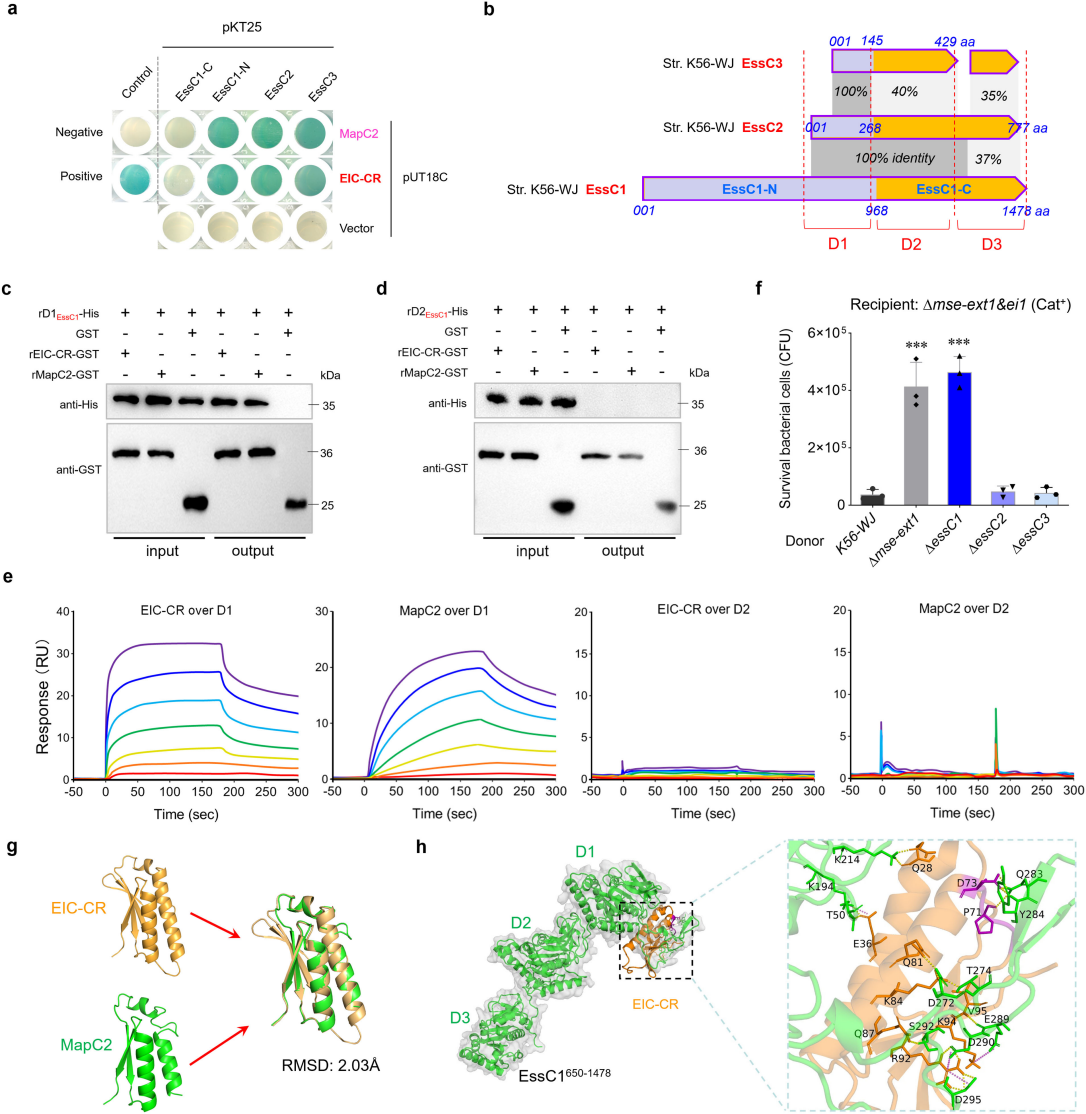
EIC-CR binds to the D1 ATPase domain of EssC. **(a)** Bacterial two-hybrid assays detect the interactions of EIC-CR and MapC2 with the EssC-related fragments. Assays were performed using the BACTH (Bacterial Adenylate Cyclase Two-Hybrid) System kit (Euromedex, France) according to the supplier’s instructions. The bacterial cells transformed with plasmids pKT25-zip and pUT18C-zip served as positive controls, and those with pKT25 and pUT18C served as negative controls. A blue appearance suggested a positive result. Similar results have been verified in two other independent experiments. **(b)** Schematic representation of the full-length EssC1, truncated EssC2, and truncated EssC3 encoded within the T7SSb locus of strain K56-WJ. **(c)** Pull-down assay confirmed the direct interaction of EIC-CR and MapC2 with the D1 ATPase domain. The assays were performed as described in the **Figure 3c** legend. **(d)** Pull-down assay detected the interaction between EIC-CR/MapC2 and the D2 ATPase domain of EssC1. **(e)** SPR sensorgram for EIC-CR/MapC2 analyte (twofold dilutions; 4–256 nM) binding to immobilized D1/D2 ATPase ligand. SPR responses are indicated in resonance units (RU). **(f)** Effects of EssC1, EssC2, and EssC3 on interbacterial competition. The assays were performed as outlined in the procedure of **Figure 1d**. **(g)** Ribbon depiction showing the superimposition of EIC-CR and MapC2. The structures of EIC-CR and MapC2 were predicted using AlphaFold3 with confidence levels >90% (**Supplementary Figure S8**). **(h)** Docked conformation and hydrogen bond interaction map of EssC1^650–1478^ (shown in green) to EIC-CR (shown in orange). Residues critical for ligand binding are shown as orange sticks (EIC-CR) and green sticks (D1 domain), highlighting hydrogen bonding interactions. The “YxxxD” motif was labeled using purple sticks. ***P<0.001.

It should be noted that EIC-CR shares a nearly identical sequence of ~50 aa with MapC2 in diverse *S. suis* strains, including the presence of a “YxxxD/E” motif (**Figure 4e**), suggesting a potential involvement with the EssC activation. The findings of the two-hybrid analyses, as depicted in **Figure 5a**, indicate that three fragments related to EssC (EssC1-N, truncated EssC2, and EssC3) are bound by EIC-CR and MapC2 during the co-incubation. In strain K56-WJ, the truncated EssC2 and EssC3 variants are located downstream of the full-length EssC1 (**Figure 1a**). All typical MSE-ExT effectors are situated downstream of the EssC3 variant and are, thus, categorized as EssC3-specific effectors (**Figure 3a**). Multiple sequence alignment showed that all three EssCs in strain K56-WJ contain the identical D1 ATPase domain (truncated in EssC2 and EssC3), while carrying distinct D2 and D3 domains (**Figure 5b**). EssC1 and EssC2 share a common D2 domain, which significantly differs from the allelic sequence of the EssC3 variant. The D1 domain is the only region that overlaps among the EssC1-N fragment, and truncated EssC2 and EssC3, and thus was hypothesized to facilitate interactions with MapC2 (YxxxD) or EIC-CR. Pull-down analysis verified that MapC2 and EIC-CR exhibited specific binding to the D1 domain of EssC1 (**Figure 5c**) but did not demonstrate an interaction with the D2 domain (**Figure 5d**). SPR analyses further confirmed the binding affinities of MapC2/EIC-CR to the D1 domain. As shown in **Figure 5e**, the relative SPR RUs were induced by MapC2 and EIC-CR in a dose-dependent manner, when the chips were immobilized with the D1 but not the D2 domain. Otherwise, deletion of neither *essC2* nor *essC3* could diminish the lethality to the recipient strain conferred by the MSE-ExT1 toxin in the competitive survival assay (**Figure 5f**). Only the depletion of EssC1 by gene deletion hindered the killing of recipient strain Δ*mse-ext1&ei1* to a level comparable to that of the Δ*mse-ext1* strain, suggesting that the export of MSE-ExT1 may significantly relate with the EIC-CR/MapC2 binding to the D1 ATPase domain of EssC1. Based on these findings, coupled with the high sequence identity observed between MapC2 and EIC-CR, the structures of these two proteins were predicted using AlphaFold3 with confidence levels >90% (**Supplementary Figure S8**) and showed a coincident superimposition (RMSD: 2.03Å), revealing a potential high degree of functional similarity between these two proteins (**Figure 5g**). The AlphaFold-predicted structures of EIC-CR and the D1-D3_EssC1_ fragment (EssC1^650-1478^) were then imported into the Cluspro 2.0 sever for molecular docking analysis, which showed a robust binding affinity between EIC-CR and the D1 domain (**Figure 5h**). We observed that 21-aa residues of the ligand EIC-CR (102 aa), including the “YxxxD” signal peptide, were able to enter the putative-binding pocket of the D1 ATPase domain and form an intensive network of hydrogen bonds. Similarly, the ligand MapC2 also docked in the pocket of D1 domain, but the binding mode showed a few differences compared with that of the ligand EIC-CR (**Supplementary Figure S9**). All these findings suggested the role of MapC2 (YxxxD) and EIC-CR in activating EssC and exporting effectors by binding to the D1 ATPase domain of EssC1.

## Discussion

Since the discovery of T7SS, substantial efforts have been made to understand its delivery mechanisms and physiological functions (Unnikrishnan et al., 2017; Wang et al., 2020; Rivera-Calzada et al., 2021; Spencer and Doran, 2022). Although numerous effectors derived from WXG, YeeF/LXG, PE and PPE proteins have been identified in previous studies (Zhang et al., 2012; Bowran and Palmer, 2021; Kobayashi, 2021), the scope of effectors awaiting discovery is likely vast, including novel types such as TslA with a unique reverse domain arrangement (Garrett et al., 2023). The findings of our study reveal MSE-ExTs as a distinctive category of T7SSb effectors in *S. suis*. Although a few ones fuse with an N-terminal YeeF domain (YeeF-MSE-ExTs) and appear alongside a cognate EssC, most of them (MSE-ExTs, 67.39%) are encoded downstream of a truncated EssC variant. Our data verified the role of MSE-ExT1 in mediating interbacterial antagonism, including killing several ExT1-sensitive isolates from the microbiota of pig tonsil, suggesting that this toxic effector may facilitate *S. suis* to obtain a competitive advantage over the specific members of the tonsil microbiota. Although the ExT domains from *S. suis* were classified into 13 distinct clades, most of them were predicted to belong to a superfamily of NAD^+^ utilizing toxins. As we know, NAD^+^ is a major cofactor in redox reactions in all life-forms, which is vital to ensure cellular homeostasis (Ren et al., 2022; Lin and Yu, 2023). Thus, bacterial NAD^+^-targeting toxins have become an emerging pathogenesis strategy, modifying the metabolic balance of the host or other competitors by consuming NAD^+^, thus acquiring survival advantage in niche colonization and bacterial lifestyle. In *Streptococcus pyogenes*, NADase (SPN) depletes the host cell energy supply by a substantial reduction of cellular NAD^+^ and ATP levels, thus promoting the apoptosis of macrophages, inhibiting the bactericidal actions of neutrophils and leukocytes, and strengthening cellular survival ability (Zhu et al., 2017; Hsieh et al., 2018). However, whether the NAD^+^-utilizing toxins delivered via T7SS exert critical roles in virulence or immune evasion for the *S. suis* or other pathogenic bacteria in the host remains to be determined.

The typical activation of T7SSb apparatus via the WXG100-like small helical proteins has been reported in diverse bacteria (Mietrach et al., 2020; Klein et al., 2022; Yang et al., 2023). Unexpectedly, cognate WXG100-like proteins are absent upstream of typical MSE-ExTs, suggesting that the translocation of MSE-ExT effectors into recipient cells remains inexplicable. We demonstrated that the secretion of typical MSE-ExTs can be activated by the interaction of EIC-CR with the conserved D1 ATPase domain of the non-neighboring full-length EssC. EIC-CR, a small helical protein-like sequence containing a conserved DUF6572 domain, is appended to the C-termini of immunity proteins, with a phenomenon previously unreported in effector recruitment to the secretion system complex. EIC-CR sequences are highly conserved among different immunity proteins, while fused with significantly divergent N-terminal fragments, which are responsible for the specific neutralization to the corresponding toxic effectors in avoiding self-intoxication. Therefore, the conserved EIC-CR domain may be a potential vaccine target to relieve the pathogenic potentials of diverse MSE-ExT effectors and T7SSb systems.

In T7SSa, it is widely acknowledged that the multimerization of EccC and the activation of ATPase activity are essential for opening the translocation channel (Rivera-Calzada et al., 2021). However, the precise mechanism by which effectors are recruited to the T7SSb apparatus remains incompletely understood. EssC is an EccC homolog, and its variants from diverse T7SSb showed a similar domain architecture, comprising N-terminal FHA1–FHA2 domains, two middle transmembrane regions, and C-terminal DUF–D1–D2–D3 domains. Currently, two pathways have been proposed to activate the ATPase activity of EssC: (1) WXG100 or WXG100-like small helical proteins play an adaptor-like role to recruit cognate LXG or LXG-like effectors to EssC hexamer, subsequently activating the ATPase activity by interacting with the D2 or D3 ATPase domain (Mietrach et al., 2020; Klein et al., 2022; Yang et al., 2023); (2) the LXG domain interacts with the EssB pseudokinase domain, which then activates the ATPase activity by interacting with two FHA domains (Tassinari et al., 2022). In *B. subtilis*, the D2 and D3 ATPase activities are reported to be dispensable for secretion via the second pathway (Ramsdell et al., 2015), suggesting that the relatively adjacent DUF or D1 ATP domain may be activated by FHA domains. In *S. suis*, most MSE-ExTs lack an N-terminal YeeF (LXG-like) domain, indicating that the second pathway is not applicable to activate the T7SSb apparatus in this study. Additionally, the small helical protein MapC2 upstream of the typical MSE-ExT could not interact with the D2 ATPase domain of truncated or untruncated cognate EssC, implying that the first pathway cannot be activated as well. Unexpectedly, MapC2 and the conserved EIC-CR domain of immunity proteins harbor a “YxxxD” signal peptide, which binds to the full-length EssC by targeting the conserved D1 ATPase domain in *S. suis* and then activates the T7SSb apparatus to dispatch MSE-ExT effectors.

The T7SSb core component EssC and its truncated duplications with exchangeable C-termini are broadly encoded downstream of a full-length EssC (Bowran and Palmer, 2021). In *S. suis*, a single T7SSb locus can also encode multiple EssC duplications of divergent lengths. These observations suggest that genetic rearrangements may frequently occur in the downstream regions of *essC* in T7SSb loci, potentially leading to a significant number of mutations, including the introduction of termination codons that interrupt the encoding genes (Reams et al., 2010; Näsvall et al., 2012). It is reasonable to deduce that these truncated duplications may originate from these genetic rearrangements, although their biological functions remain largely unclear. Several earlier studies (Warne et al., 2016; Jäger et al., 2018; Bowran and Palmer, 2021) have proposed that a full-length EssC is required for the recognition and secretion of downstream cognate effectors. In most T7SSb, the N-terminal regions of EssCs from FHA1 to D1-ATPase domains are conserved, while the D2 and D3 ATPase domains from different EssC variants exhibit significant sequence differences (Zhang et al., 2011; Ting et al., 2018; Klein et al., 2022), thus requiring binding by cognate WXG100-like or specific effector proteins for activation. For the MSE-ExT-EI pairs, the widely shared MapC2 (YxxxD) and EIC-CR are deployed to interact and activate the conserved D1 ATPase domain of the non-neighboring full-length EssC1, enabling the successful secretion of MSE-ExTs even in the absence of a cognate EssC variant. Further analysis found that the *mse-ext&eic-CR* loci are prevalent in diverse Gram-positive bacteria, with a notable presence in *Streptococcus*, *Enterococcus*, and *Bacillus* species (**Supplementary Figure S10**). However, most of them are located downstream of a full-length *essC* and *mapBC1&yapABC* loci (lacking the *mapC2* gene) in *Streptococcus* and *Enterococcus* species, suggesting the redundancy of typical and EIC-CR-mediated alternative pathways in these bacteria. In numerous *Bacillus* species, the cognate full-length *essC* gene is absent in the upstream region of *mapBC1&yapABC-mse-ext&eic-CR* loci (**Supplementary Figure S10**), implying that the EIC-CR-mediated non-canonical pathway is required for the MSE-ExT effectors’ delivery in these bacteria.

Here, MapC2 and EIC-CR appear to exhibit functional redundancy, as they both facilitate effector export. The redundancy is also present among multiple small helical partner proteins. In *S. aureus*, a total of three WXG100-like proteins (EsxB, EsxC, and EsxD) are encoded upstream of the effector EsaD, and the deletion of *esxD* (EsxD with a “YxxxE” targeting signal) resulted in the greatest degree of attenuation for EsaD secretion, while the deletion of any individual *esx* gene could not completely abrogate the effector export (Yang et al., 2023). Analogous to the effector EsaD, almost all LXG family effectors are associated with multiple WXG100-like proteins, suggesting that multiple recruitment is widely deployed in T7SSb systems, while their precise roles during effector secretion require further validation. The small partner proteins, also designated as adaptors in the type VI secretion system (T6SS), have been reported to be redundantly deployed. For instance, a type of C-terminal extension in VgrG proteins consists of a DUF2345 domain and a transthyretin-like (TTR) domain, and both of them are involved in recruiting effectors to the T6SS apparatus (Cianfanelli et al., 2016; Quentin et al., 2018). This strategy of deploying multiple partners/adaptors in effector–immunity pairs effectively mitigates the risks of effector secretion being interrupted by frequent sequence mutations during genetic recombination or protease degradation during intracellular transport.

**Figure 6:**
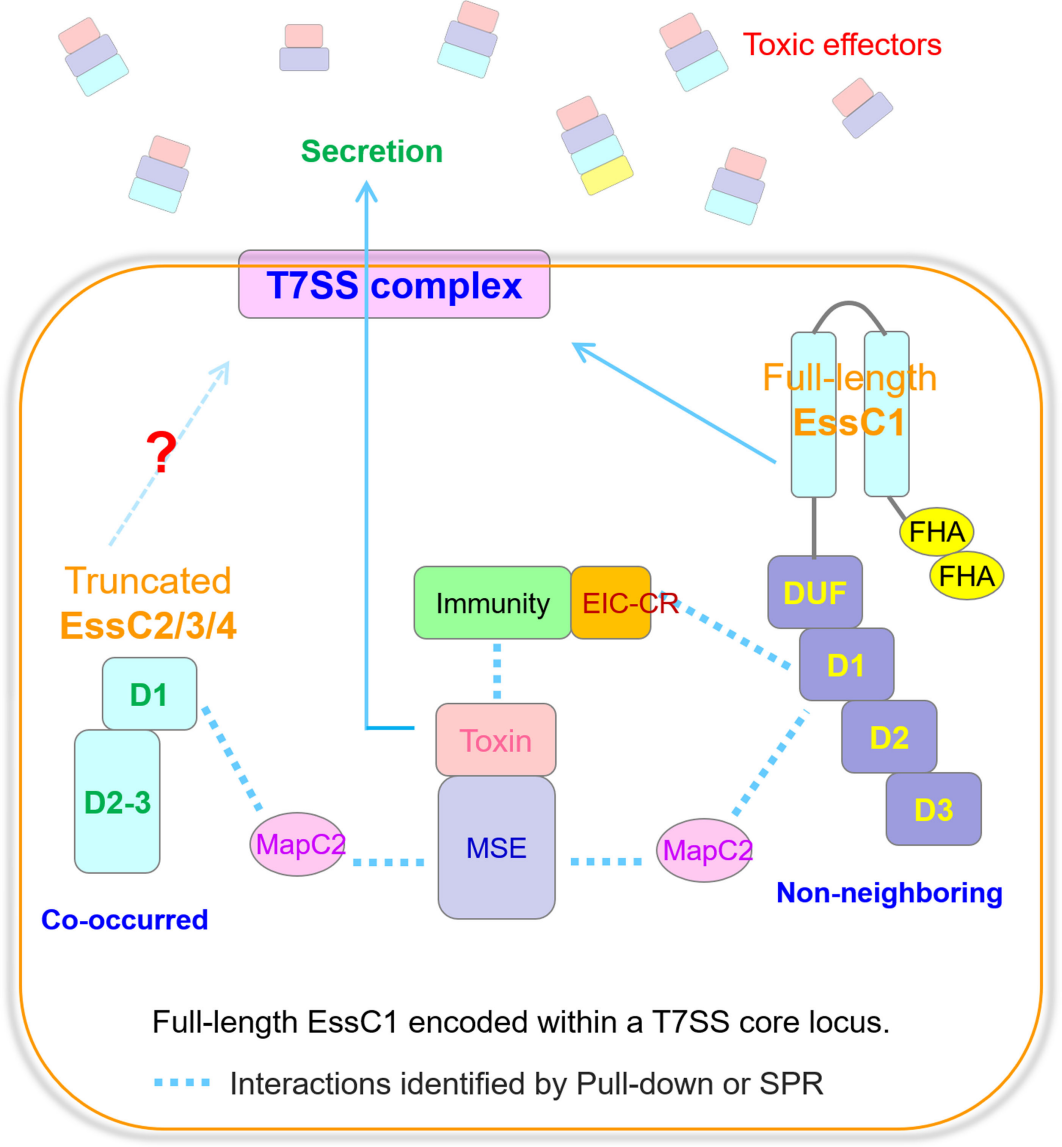
The delivery pathways of MSE-ExTs in *S. suis*. Model depicting the recruitment patterns of MSE-ExT effectors for EssC activation. The MSE-ExT effectors are recruited by two pathways, namely, (1) MapC2 interacting with MSE and activating the non-neighboring EssC variant encoded by the T7SS core locus, and (2) EIC-CR of the immunity–toxin complex activating the non-neighboring EssC variant; finally, the effectors are exported via the T7SSb channel. Currently, there is no study identifying whether the truncated EssCs can interact with another EssC molecule and assemble into the T7SSb complex when their transmembrane region is absent.

In summary, we have delineated an example of a novel T7SSb substrate encoded downstream of a truncated cognate EssC variant in *S. suis* and propose a model (**Figure 6**) in which effectors are recruited either by MapC2 through the MSE interaction or by EIC-CR through a toxin–immunity binding mechanism to the T7SSb apparatus, which then activates the D1 ATPase domain of the full-length EssC encoded in the core T7SSb locus that is activated by either MapC2 or EIC-CR and triggers the secretion of MSE-ExTs. Moving forward, it will be a promising opportunity to identify further examples that are also encoded within incomplete *essC* loci that have undergone frequent genetic rearrangement and to share this alternative pathway to ensure the delivery of effectors.

## Data Availability

The datasets presented in this study can be found in online repositories. The names of the repository/repositories and accession number(s) can be found below: Figshare (https://doi.org/10.6084/m9.figshare.29487953).

## References

[B1] Abdallah A. M. Gey van Pittius N. C. Champion P. A. D. Cox J. Luirink J. Vandenbroucke-Grauls C. M. J. E.. (2007). Type VII secretion–mycobacteria show the way. Nat. Rev. Microbiol. 5, 883–891. doi: 10.1038/nrmicro1773, PMID: 17922044

[B2] Bowman L. Palmer T. (2021). The type VII secretion system of staphylococcus. Annu. Rev. Microbiol. 75, 471–494. doi: 10.1146/annurev-micro-012721-123600, PMID: 34343022 PMC7619418

[B3] Bowran K. Palmer T. (2021). Extreme genetic diversity in the type VII secretion system of Listeria monocytogenes suggests a role in bacterial antagonism. Microbiol. (Reading) 167, mic.0.001034. doi: 10.1099/mic.0.001034, PMID: 33599605 PMC7613182

[B4] Camacho C. Coulouris G. Avagyan V. Ma N. Papadopoulos J. Bealer K.. (2009). BLAST+: architecture and applications. BMC Bioinf. 10, 421. doi: 10.1186/1471-2105-10-421, PMID: 20003500 PMC2803857

[B5] Cao Z. Casabona M. G. Kneuper H. Chalmers J. D. Palmer T. (2016). The type VII secretion system of Staphylococcus aureus secretes a nuclease toxin that targets competitor bacteria. Nat. Microbiol. 2, 16183. doi: 10.1038/nmicrobiol.2016.183, PMID: 27723728 PMC5325307

[B6] Champion P. A. D. Stanley S. A. Champion M. M. Brown E. J. Cox J. S. (2006). C-terminal signal sequence promotes virulence factor secretion in Mycobacterium tuberculosis. Science 313, 1632–1636. doi: 10.1126/science.1131167, PMID: 16973880

[B7] Cianfanelli F. R. Alcoforado Diniz J. Guo M. De Cesare V. Trost M. Coulthurst S. J. (2016). VgrG and PAAR proteins define distinct versions of a functional type VI secretion system. PloS Pathog. 12, e1005735. doi: 10.1371/journal.ppat.1005735, PMID: 27352036 PMC4924876

[B8] Daleke M. H. Ummels R. Bawono P. Heringa J. Vandenbroucke-Grauls C. M. J. E. Luirink J.. (2012). General secretion signal for the mycobacterial type VII secretion pathway. Proc. Natl. Acad. Sci. U.S.A. 109, 11342–11347. doi: 10.1073/pnas.1119453109, PMID: 22733768 PMC3396530

[B9] Dedrick R. M. Aull H. G. Jacobs-Sera D. Garlena R. A. Russell D. A. Smith B. E.. (2021). The prophage and plasmid mobilome as a likely driver of mycobacterium abscessus diversity. mBio 12, e03441–e03420. doi: 10.1128/mBio.03441-20, PMID: 33785627 PMC8092301

[B10] Feng Y. Zhang H. Ma Y. Gao G. F. (2010). Uncovering newly emerging variants of Streptococcus suis, an important zoonotic agent. Trends Microbiol. 18, 124–131. doi: 10.1016/j.tim.2009.12.003, PMID: 20071175

[B11] Garrett S. R. Mietrach N. Deme J. Bitzer A. Yang Y. Ulhuq F. R.. (2023). A type VII-secreted lipase toxin with reverse domain arrangement. Nat. Commun. 14, 8438. doi: 10.1038/s41467-023-44221-y, PMID: 38114483 PMC10730906

[B12] Gottschalk M. Xu J. Calzas C. Segura M. (2010). Streptococcus suis: a new emerging or an old neglected zoonotic pathogen? Future Microbiol. 5, 371–391. doi: 10.2217/fmb.10.2, PMID: 20210549

[B13] Goyette-Desjardins G. Auger J.-P. Xu J. Segura M. Gottschalk M. (2014). Streptococcus suis, an important pig pathogen and emerging zoonotic agent-an update on the worldwide distribution based on serotyping and sequence typing. Emerg. Microbes Infect. 3, e45. doi: 10.1038/emi.2014.45, PMID: 26038745 PMC4078792

[B14] Green E. R. Mecsas J. (2016). Bacterial secretion systems: an overview. Microbiol. Spectr. 4, 10.1128/microbiolspec.vmbf-0012–2015. doi: 10.1128/microbiolspec.VMBF-0012-2015, PMID: 26999395 PMC4804464

[B15] Gu Q. Zhu X. Yu Y. Jiang T. Pan Z. Ma J.. (2024). Type II and IV toxin-antitoxin systems coordinately stabilize the integrative and conjugative element of the ICESa2603 family conferring multiple drug resistance in Streptococcus suis. PloS Pathog. 20, e1012169. doi: 10.1371/journal.ppat.1012169, PMID: 38640137 PMC11062541

[B16] Hsieh C.-L. Huang H.-M. Hsieh S.-Y. Zheng P.-X. Lin Y.-S. Chiang-Ni C.. (2018). NAD-glycohydrolase depletes intracellular NAD+ and inhibits acidification of autophagosomes to enhance multiplication of group A streptococcus in endothelial cells. Front. Microbiol. 9. doi: 10.3389/fmicb.2018.01733, PMID: 30123194 PMC6085451

[B17] Hsu T. Hingley-Wilson S. M. Chen B. Chen M. Dai A. Z. Morin P. M.. (2003). The primary mechanism of attenuation of bacillus Calmette-Guerin is a loss of secreted lytic function required for invasion of lung interstitial tissue. Proc. Natl. Acad. Sci. United States America 100, 12420–25. doi: 10.1073/pnas.1635213100, PMID: 14557547 PMC218773

[B18] Jäger F. Kneuper H. Palmer T. (2018). EssC is a specificity determinant for Staphylococcus aureus type VII secretion. Microbiol. (Reading England) 164, 816–820. doi: 10.1099/mic.0.000650, PMID: 29620499 PMC5994694

[B19] Jamet A. Nassif X. (2015). New players in the toxin field: polymorphic toxin systems in bacteria. mBio 6, 10.1128/mbio.00285-15. doi: 10.1128/mBio.00285-15, PMID: 25944858 PMC4436062

[B20] Jumper J. Evans R. Pritzel A. Green T. Figurnov M. Ronneberger O.. (2021). Highly accurate protein structure prediction with AlphaFold. Nature 596, 583–589. doi: 10.1038/s41586-021-03819-2, PMID: 34265844 PMC8371605

[B21] Kaundal S. Deep A. Kaur G. Thakur K. G. (2020). Molecular and biochemical characterization of yeeF/yezG, a polymorphic toxin-immunity protein pair from bacillus subtilis. Front. Microbiol. 11. doi: 10.3389/fmicb.2020.00095, PMID: 32117125 PMC7033585

[B22] Klein T. A. Ahmad S. Whitney J. C. (2020). Contact-dependent interbacterial antagonism mediated by protein secretion machines. Trends Microbiol. 28, 387–400. doi: 10.1016/j.tim.2020.01.003, PMID: 32298616

[B23] Klein T. A. Grebenc D. W. Shah P. Y. McArthur O. D. Dickson B. H. Surette M. G.. (2022). Dual targeting factors are required for LXG toxin export by the bacterial type VIIb secretion system. mBio 13, e0213722. doi: 10.1128/mbio.02137-22, PMID: 36036513 PMC9600955

[B24] Klein T. A. Shah P. Y. Gkragkopoulou P. Grebenc D. W. Kim Y. Whitney J. C. (2024). Structure of a tripartite protein complex that targets toxins to the type VII secretion system. Proc. Natl. Acad. Sci. United States America 121, e2312455121. doi: 10.1073/pnas.2312455121, PMID: 38194450 PMC10801868

[B25] Kneuper H. Cao Z. P. Twomey K. B. Zoltner M. Jäger F. Cargill J. S.. (2014). Heterogeneity in ess transcriptional organization and variable contribution of the Ess/Type VII protein secretion system to virulence across closely related Staphylocccus aureus strains. Mol. Microbiol. 93, 928–943. doi: 10.1111/mmi.12707, PMID: 25040609 PMC4285178

[B26] Kobayashi K. (2021). Diverse LXG toxin and antitoxin systems specifically mediate intraspecies competition in Bacillus subtilis biofilms. PloS Genet. 17, e1009682. doi: 10.1371/journal.pgen.1009682, PMID: 34280190 PMC8321402

[B27] Kozakov D. Hall D. R. Xia B. Porter K. A. Padhorny D. Yueh C.. (2017). The ClusPro web server for protein-protein docking. Nat. Protoc. 12, 255–278. doi: 10.1038/nprot.2016.169, PMID: 28079879 PMC5540229

[B28] Lai L. Dai J. Tang H. Zhang S. Wu C. Qiu W.. (2017). Streptococcus suis serotype 9 strain GZ0565 contains a type VII secretion system putative substrate EsxA that contributes to bacterial virulence and a vanZ-like gene that confers resistance to teicoplanin and dalbavancin in Streptococcus agalactiae. Vet. Microbiol. 205, 26–33. doi: 10.1016/j.vetmic.2017.04.030, PMID: 28622857

[B29] Letunic I. Bork P. (2021). Interactive Tree Of Life (iTOL) v5: an online tool for phylogenetic tree display and annotation. Nucleic Acids Res. 49, W293–W296. doi: 10.1093/nar/gkab301, PMID: 33885785 PMC8265157

[B30] Liang Z. Wu H. Bian C. Chen H. Shen Y. Gao X.. (2022). The antimicrobial systems of Streptococcus suis promote niche competition in pig tonsils. Virulence 13, 781–793. doi: 10.1080/21505594.2022.2069390, PMID: 35481413 PMC9067509

[B31] Lin R. Yu J. (2023). The role of NAD+ metabolism in macrophages in age-related macular degeneration. Mech. Ageing Dev. 209, 111755. doi: 10.1016/j.mad.2022.111755, PMID: 36435209

[B32] Ma J. Pan Z. Huang J. Sun M. Lu C. Yao H. (2017). The Hcp proteins fused with diverse extended-toxin domains represent a novel pattern of antibacterial effectors in type VI secretion systems. Virulence 8, 1189–1202. doi: 10.1080/21505594.2017.1279374, PMID: 28060574 PMC5711352

[B33] Mietrach N. Damián-Aparicio D. Mielich-Süss B. Lopez D. Geibel S. (2020). Substrate interaction with the essC coupling protein of the type VIIb secretion system. J. Bacteriol 202, e00646-19. doi: 10.1128/JB.00646-19, PMID: 31964696 PMC7167477

[B34] Mj P. (2002). The ESAT-6/WXG100 superfamily – and a new Gram-positive secretion system? Trends Microbiol. 10, 209–212. doi: 10.1016/s0966-842x(02)02345-4, PMID: 11973144

[B35] Näsvall J. Sun L. Roth J. R. Andersson D. I. (2012). Real-time evolution of new genes by innovation, amplification, and divergence. Science 338, 384–387. doi: 10.1126/science.1226521, PMID: 23087246 PMC4392837

[B36] Pei J. Tang M. Grishin N. V. (2008). PROMALS3D web server for accurate multiple protein sequence and structure alignments. Nucleic Acids Res. 36, W30–W34. doi: 10.1093/nar/gkn322, PMID: 18503087 PMC2447800

[B37] Powell H. R. Islam S. A. David A. Sternberg M. J. E. (2025). Phyre2.2: A community resource for template-based protein structure prediction. J. Mol. Biol. 437, 168960. doi: 10.1016/j.jmb.2025.168960, PMID: 40133783 PMC7617537

[B38] Pym A. S. Brodin P. Majlessi L. Brosch R. Demangel C. Williams A.. (2003). Recombinant BCG exporting ESAT-6 confers enhanced protection against tuberculosis. Nat. Med. 9, 533–539. doi: 10.1038/nm859, PMID: 12692540

[B39] Quentin D. Ahmad S. Shanthamoorthy P. Mougous J. D. Whitney J. C. Raunser S. (2018). Mechanism of loading and translocation of type VI secretion system effector Tse6. Nat. Microbiol. 3, 1142–1152. doi: 10.1038/s41564-018-0238-z, PMID: 30177742 PMC6488228

[B40] Ramsdell T. L. Huppert L. A. Sysoeva T. A. Fortune S. M. Burton B. M. (2015). Linked domain architectures allow for specialization of function in the FtsK/SpoIIIE ATPases of ESX secretion systems. J. Mol. Biol. 427, 1119–1132. doi: 10.1016/j.jmb.2014.06.013, PMID: 24979678 PMC4277743

[B41] Reams A. B. Kofoid E. Savageau M. Roth J. R. (2010). Duplication frequency in a population of Salmonella enterica rapidly approaches steady state with or without recombination. Genetics 184, 1077–1094. doi: 10.1534/genetics.109.111963, PMID: 20083614 PMC2865909

[B42] Ren Z. Xu Y. Li T. Sun W. Tang Z. Wang Y.. (2022). NAD+ and its possible role in gut microbiota: Insights on the mechanisms by which gut microbes influence host metabolism. Anim. Nutr. 10, 360–371. doi: 10.1016/j.aninu.2022.06.009, PMID: 35949199 PMC9356074

[B43] Renshaw P. S. Lightbody K. L. Veverka V. Muskett F. W. Kelly G. Frenkiel T. A.. (2005). Structure and function of the complex formed by the tuberculosis virulence factors CFP-10 and ESAT-6. EMBO J. 24, 2491–2498. doi: 10.1038/sj.emboj.7600732, PMID: 15973432 PMC1176459

[B44] Rivera-Calzada A. Famelis N. Llorca O. Geibel S. (2021). Type VII secretion systems: structure, functions and transport models. Nat. Rev. Microbiol. 19, 567–584. doi: 10.1038/s41579-021-00560-5, PMID: 34040228

[B45] Roussin M. Salcedo S. P. (2021). NAD+-targeting by bacteria: an emerging weapon in pathogenesis. FEMS Microbiol. Rev. 45, fuab037. doi: 10.1093/femsre/fuab037, PMID: 34223888

[B46] Schrödinger L. L. C. (2015). The pyMOL molecular graphics system, version 1.8. Schrödinger L. L. C., New York, NY, USA.

[B47] Segura M. (2020). Streptococcus suis research: progress and challenges. Pathog. (Basel Switzerland) 9, 707. doi: 10.3390/pathogens9090707, PMID: 32867188 PMC7557840

[B48] Spencer B. L. Doran K. S. (2022). Evolving understanding of the type VII secretion system in Gram-positive bacteria. PloS Pathog. 18, e1010680. doi: 10.1371/journal.ppat.1010680, PMID: 35901012 PMC9333272

[B49] Spencer B. L. Tak U. Mendonça J. C. Nagao P. E. Niederweis M. Doran K. S. (2021). A type VII secretion system in Group B Streptococcus mediates cytotoxicity and virulence. PloS Pathog. 17, e1010121. doi: 10.1371/journal.ppat.1010121, PMID: 34871327 PMC8675928

[B50] Stanley S. A. Raghavan S. Hwang W. W. Cox J. S. (2003). Acute infection and macrophage subversion by Mycobacterium tuberculosis require a specialized secretion system. Proc. Natl. Acad. Sci. United States America 100, 13001–006. doi: 10.1073/pnas.2235593100, PMID: 14557536 PMC240734

[B51] Sun J. Siroy A. Lokareddy R. K. Speer A. Doornbos K. S. Cingolani G.. (2015). The tuberculosis necrotizing toxin kills macrophages by hydrolyzing NAD. Nat. Struct. Mol. Biol. 22, 672–678. doi: 10.1038/nsmb.3064, PMID: 26237511 PMC4560639

[B52] Sundaramoorthy R. Fyfe P. K. Hunter W. N. (2008). Structure of Staphylococcus aureus EsxA suggests a contribution to virulence by action as a transport chaperone and/or adaptor protein. J. Mol. Biol. 383, 603–614. doi: 10.1016/j.jmb.2008.08.047, PMID: 18773907 PMC3465917

[B53] Tak U. Dokland T. Niederweis M. (2021). Pore-forming Esx proteins mediate toxin secretion by Mycobacterium tuberculosis. Nat. Commun. 12, 394. doi: 10.1038/s41467-020-20533-1, PMID: 33452244 PMC7810871

[B54] Tan C. Zhang A. Chen H. Zhou R. (2019). Recent proceedings on prevalence and pathogenesis of streptococcus suis. Curr. Issues Mol. Biol. 32, 473–520. doi: 10.21775/cimb.032.473, PMID: 31166178

[B55] Tassinari M. Doan T. Bellinzoni M. Chabalier M. Ben-Assaya M. Martinez M.. (2022). The antibacterial type VII secretion system of bacillus subtilis: structure and interactions of the pseudokinase yukC/essB. mBio 13, e0013422. doi: 10.1128/mbio.00134-22, PMID: 36154281 PMC9600267

[B56] Ting S.-Y. Bosch D. E. Mangiameli S. M. Radey M. C. Huang S. Park Y.-J.. (2018). Bifunctional immunity proteins protect bacteria against ftsZ-targeting ADP-ribosylating toxins. Cell 175, 1380–1392.e14. doi: 10.1016/j.cell.2018.09.037, PMID: 30343895 PMC6239978

[B57] Trott O. Olson A. J. (2010). AutoDock Vina: improving the speed and accuracy of docking with a new scoring function, efficient optimization, and multithreading. J. Comput. Chem. 31, 455–61. doi: 10.1002/jcc.21334, PMID: 19499576 PMC3041641

[B58] UniProt Consortium (2019). UniProt: a worldwide hub of protein knowledge. Nucleic Acids Res. 47, D506–D515. doi: 10.1093/nar/gky1049, PMID: 30395287 PMC6323992

[B59] Unnikrishnan M. Constantinidou C. Palmer T. Pallen M. J. (2017). The enigmatic esx proteins: looking beyond mycobacteria. Trends Microbiol. 25, 192–204. doi: 10.1016/j.tim.2016.11.004, PMID: 27894646

[B60] van der Wel N. Hava D. Houben D. Fluitsma D. van Zon M. Pierson J.. (2007). M. tuberculosis and M. leprae translocate from the phagolysosome to the cytosol in myeloid cells. Cell 129, 1287–1298. doi: 10.1016/j.cell.2007.05.059, PMID: 17604718

[B61] Vötsch D. Willenborg M. Weldearegay Y. B. Valentin-Weigand P. (2018). Streptococcus suis - the “Two faces” of a pathobiont in the porcine respiratory tract. Front. Microbiol. 9. doi: 10.3389/fmicb.2018.00480, PMID: 29599763 PMC5862822

[B62] Wang S. Zhou K. Yang X. Zhang B. Zhao Y. Xiao Y.. (2020). Structural insights into substrate recognition by the type VII secretion system. Protein Cell 11, 124–137. doi: 10.1007/s13238-019-00671-z, PMID: 31758528 PMC6954902

[B63] Warne B. Harkins C. P. Harris S. R. Vatsiou A. Stanley-Wall N. Parkhill J.. (2016). The Ess/Type VII secretion system of Staphylococcus aureus shows unexpected genetic diversity. BMC Genomics 17, 222. doi: 10.1186/s12864-016-2426-7, PMID: 26969225 PMC4788903

[B64] Waterhouse A. Bertoni M. Bienert S. Studer G. Tauriello G. Gumienny R.. (2018). SWISS-MODEL: homology modelling of protein structures and complexes. Nucleic Acids Res. 46, W296–W303. doi: 10.1093/nar/gky427, PMID: 29788355 PMC6030848

[B65] Whitney J. C. Peterson S. B. Kim J. Pazos M. Verster A. J. Radey M. C.. (2017). A broadly distributed toxin family mediates contact-dependent antagonism between gram-positive bacteria. eLife 6, e26938. doi: 10.7554/eLife.26938, PMID: 28696203 PMC5555719

[B66] Wojtowicz W. M. Vielmetter J. Fernandes R. A. Siepe D. H. Eastman C. L. Chisholm G. B.. (2020). A human igSF cell-surface interactome reveals a complex network of protein-protein interactions. Cell 182, 1027–1043.e17. doi: 10.1016/j.cell.2020.07.025, PMID: 32822567 PMC7440162

[B67] Yang Y. Boardman E. Deme J. Alcock F. Lea S. Palmer T. (2023). Three small partner proteins facilitate the type VII-dependent secretion of an antibacterial nuclease. mBio 14, e0210023. doi: 10.1128/mbio.02100-23, PMID: 37815362 PMC10653861

[B68] Zhang D. de Souza R. F. Anantharaman V. Iyer L. M. Aravind L. (2012). Polymorphic toxin systems: Comprehensive characterization of trafficking modes, processing, mechanisms of action, immunity and ecology using comparative genomics. Biol. Direct 7, 18. doi: 10.1186/1745-6150-7-18, PMID: 22731697 PMC3482391

[B69] Zhang D. Iyer L. M. Aravind L. (2011). A novel immunity system for bacterial nucleic acid degrading toxins and its recruitment in various eukaryotic and DNA viral systems. Nucleic Acids Res. 39, 4532–4552. doi: 10.1093/nar/gkr036, PMID: 21306995 PMC3113570

[B70] Zhang W. Lu C. P. (2007). Immunoproteomics of extracellular proteins of Chinese virulent strains of Streptococcus suis type 2. Proteomics 7, 4468–76. doi: 10.1002/pmic.200700294, PMID: 18022935

[B71] Zhu Y. Dong W. Ma J. Zhang Y. Pan Z. Yao H. (2019). Utilization of the ComRS system for the rapid markerless deletion of chromosomal genes in Streptococcus suis. Future Microbiol. 14, 207–222. doi: 10.2217/fmb-2018-0279, PMID: 30663887

[B72] Zhu L. Olsen R. J. Lee J. D. Porter A. R. DeLeo F. R. Musser J. M. (2017). Contribution of secreted NADase and streptolysin O to the pathogenesis of epidemic serotype M1 streptococcus pyogenes infections. Am. J. Pathol. 187, 605–613. doi: 10.1016/j.ajpath.2016.11.003, PMID: 28034602 PMC5397666

