# Supplementary material for: Identifying a type of toxic effectors exported by the type VII secretion system to enhance competitive fitness in Streptococcus suis
Source: Front Cell Infect Microbiol. 2025 Oct 14;15:1685307. doi: 10.3389/fcimb.2025.1685307 (PMC12558993; doi:10.3389/fcimb.2025.1685307)
Supplement: Supplementary file 1 [file DataSheet1.pdf]

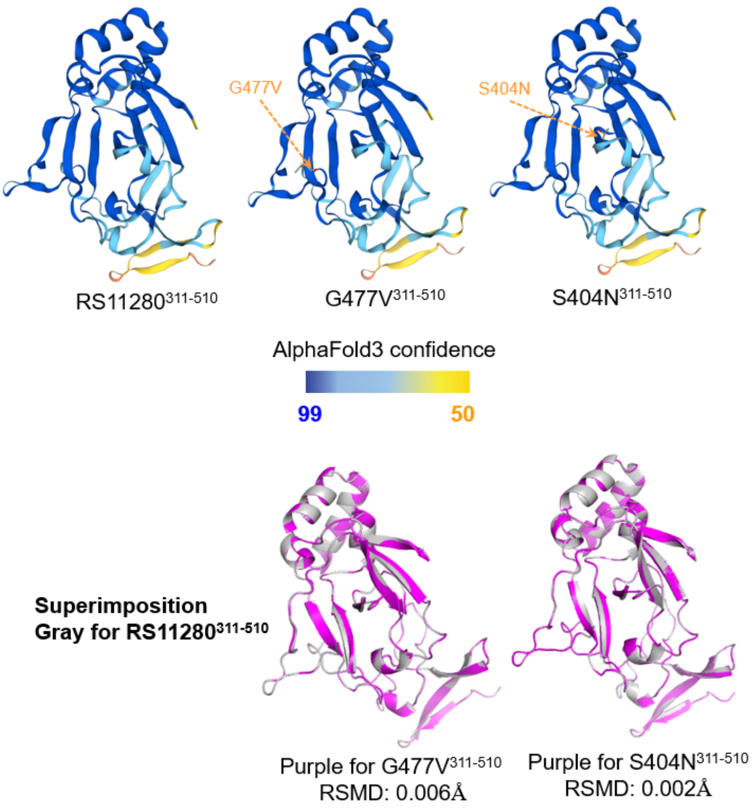

Fig. S1 AlphaFold3-predicted structures of RS11280$^{311\text{-}510}$, G477V$^{311\text{-}510}$ and S404N$^{311\text{-}510}$, and their structural superimposition.

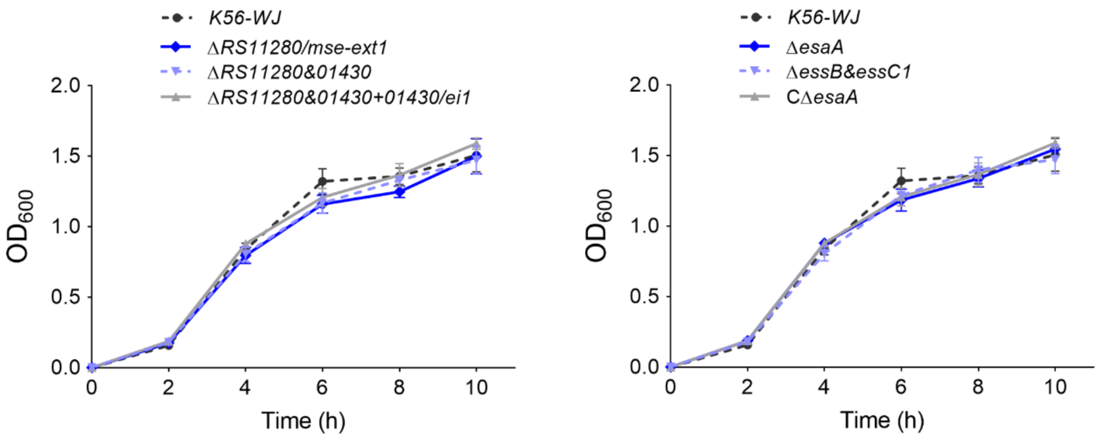

Fig. S2 The growth curve of K56-WJ and its derived mutant strains. Error bars indicate the mean±standard deviation of three biological replicates.

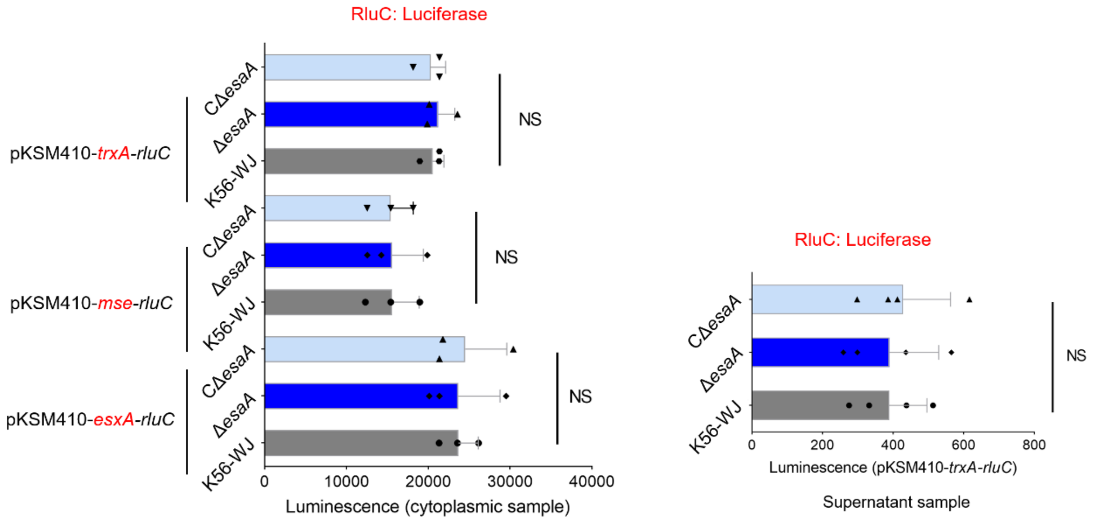

Fig. S3 RluC assays of MSE, EsxA and TrxA by detecting the total protein samples of cell pellet or supermatant protein samples in the indicated *S. suis* strains. The prepared samples of indicated strains were measured by using the Renilla Luciferase Reporter Gene Assay Kit (Beyotime). The fluorescence value of TrxA, a cytoplasmic protein, was measured and used as an internal reference. CΔ*esaA* is a complementary strain of Δ*esaA*.

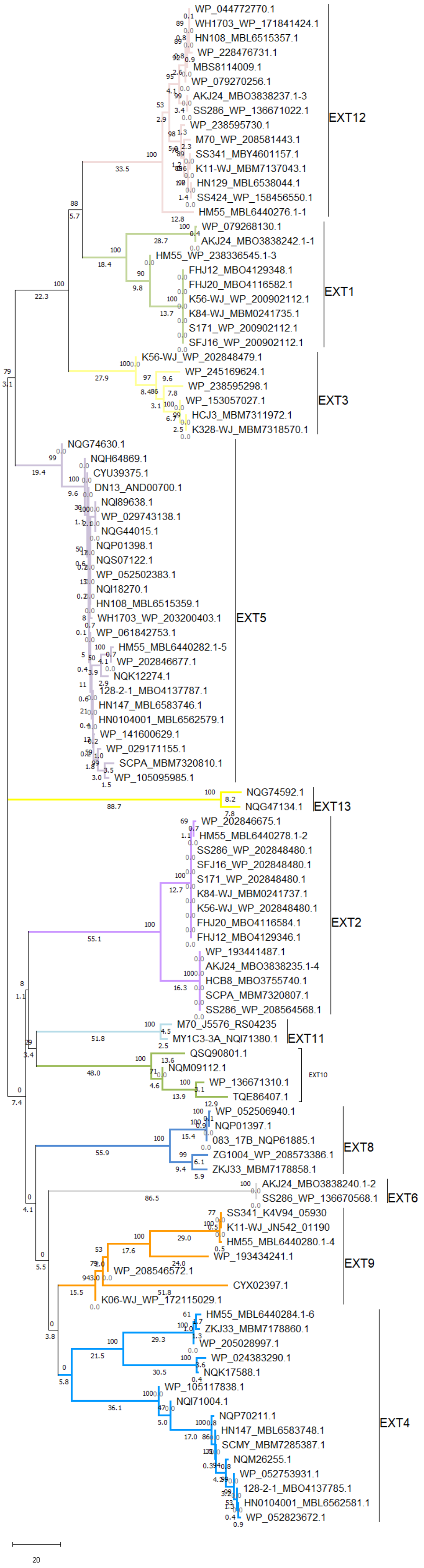

WP_044772770.1
WH1703_WP_171841424.1
HN108_MBL6515357.1
WP_228476731.1
MBS8114009.1
WP_079270256.1
AKJ24_MBO3838237.1-3
SS286_WP_136671022.1
WP_238595730.1
M70_WP_208581443.1
SS341_MBY4601157.1
K11-WJ_MBM7137043.1
HN129_MBL6538044.1
SS424_WP_158456550.1
HM55_MBL6440276.1-1
EXT12
WP_079268130.1
AKJ24_MBO3838242.1-1
HM55_WP_238336545.1-3
FHJ12_MBO4129348.1
FHJ20_MBO4116582.1
K56-WJ_WP_200902112.1
K84-WJ_MBM0241735.1
S171_WP_200902112.1
SFJ16_WP_200902112.1
EXT1
K56-WJ_WP_202848479.1
WP_245169624.1
WP_238595298.1
WP_153057027.1
HCJ3_MBM7311972.1
K328-WJ_MBM7318570.1
EXT3
NQG74630.1
NQH64869.1
CYU39375.1
DN13_AND00700.1
NQI89638.1
WP_029743138.1
NQG44015.1
NQP01398.1
NQS07122.1
WP_052502383.1
NQI18270.1
HN108_MBL6515359.1
WH1703_WP_203200403.1
WP_061842753.1
HM55_MBL6440282.1-5
WP_202846677.1
NQK12274.1
128-2-1_MBO4137787.1
HN147_MBL6583746.1
HN0104001_MBL6562579.1
WP_141600629.1
WP_029171155.1
SCPA_MBM7320810.1
WP_105095985.1
EXT5
NQG74592.1
NQG47134.1
EXT13
WP_202846675.1
HM55_MBL6440278.1-2
SS286_WP_202848480.1
SFJ16_WP_202848480.1
S171_WP_202848480.1
K84-WJ_MBM0241737.1
K56-WJ_WP_202848480.1
FHJ20_MBO4116584.1
FHJ12_MBO4129346.1
WP_193441487.1
AKJ24_MBO3838235.1-4
HCB8_MBO3755740.1
SCPA_MBM7320807.1
SS286_WP_208564568.1
EXT2
M70_J5576_RS04235
MY1C3-3A_NQI71380.1
EXT11
QSQ90801.1
NQM09112.1
WP_136671310.1
TQE86407.1
EXT10
WP_052506940.1
NQP01397.1
083_17B_NQP61885.1
ZG1004_WP_208573386.1
ZKJ33_MBM7178858.1
EXT8
AKJ24_MBO3838240.1-2
SS286_WP_136670568.1
EXT6
SS341_K4V94_05930
K11-WJ_JN542_01190
HM55_MBL6440280.1-4
WP_193434241.1
WP_208546572.1
CYX02397.1
K06-WJ_WP_172115029.1
EXT9
HM55_MBL6440284.1-6
ZKJ33_MBM7178860.1
WP_205028997.1
WP_024383290.1
NQK17588.1
WP_105117838.1
NQI71004.1
NQP70211.1
HN147_MBL6583748.1
SCMY_MBM7285387.1
NQM26255.1
WP_052753931.1
128-2-1_MBO4137785.1
HN0104001_MBL6562581.1
WP_052823672.1
EXT4
20

Fig. S4 Phylogenetic analysis of 104 randomly selected MSE-ExT effectors wildly encoded in S. suis isolates. A neighbor-joining tree was constructed based on the C-terminal extension of MSE-ExT effectors using the MEGA software version 7.0.

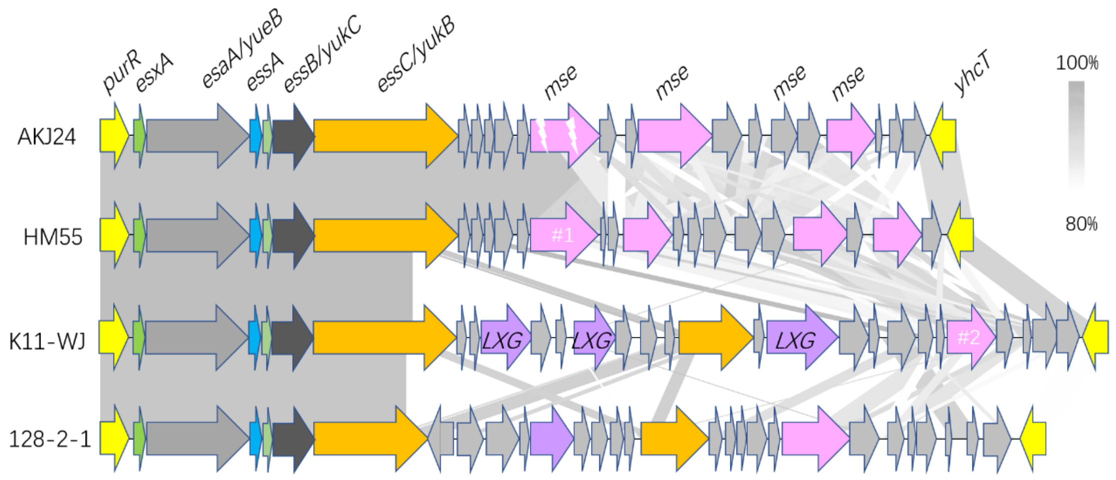

Fig. S5 Graphical depiction of four representative T7SSb locis harbouring diverse MSE related effector-immunity pairs in *S. suis*. Four *S. suis* strains, AKJ24, HM55, K11-WJ and 128-2-1, were selected for genetic neighborhood analysis, which showed that a single T7SSb locus may encode multiple MSE duplications with exchangeable ExTs. The direction of the arrows indicates the direction of transcription. #1 ORF was revised from 159024 to 161126 of HM55 genome Scaffold6, and #2 ORF was revised from 235637 to 236998 of K11-WJ genome Scaffold1.

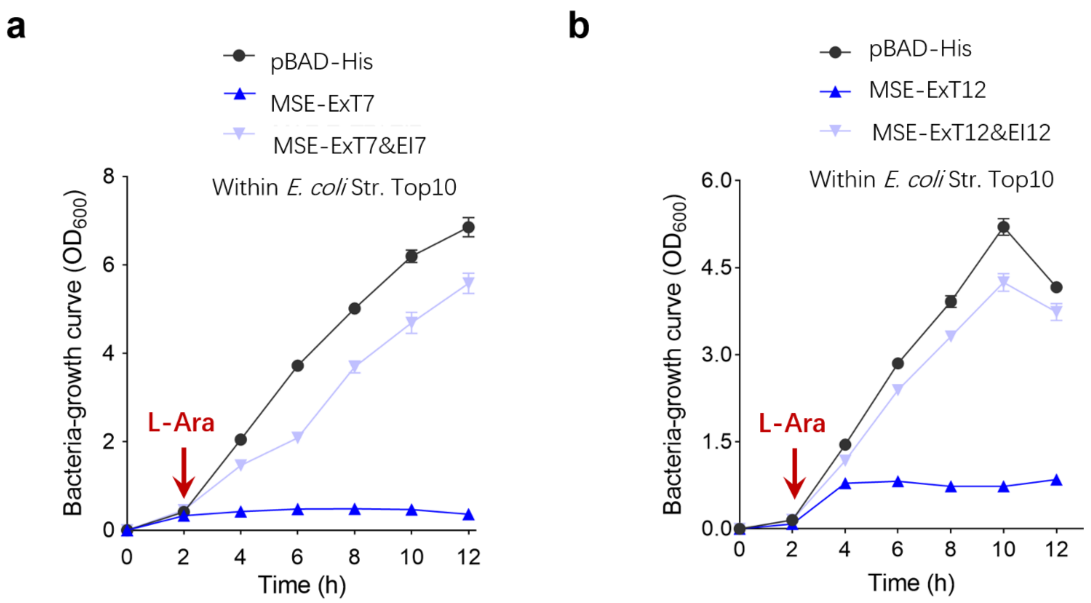

Fig. S6 Identification of the antibacterial activities of MSE-ExT7 and -ExT12. Growth curves of *E. coli* cells expressing the indicated proteins. The cultures were induced by L-arabinose at the indicated time (shown by the red arrow).

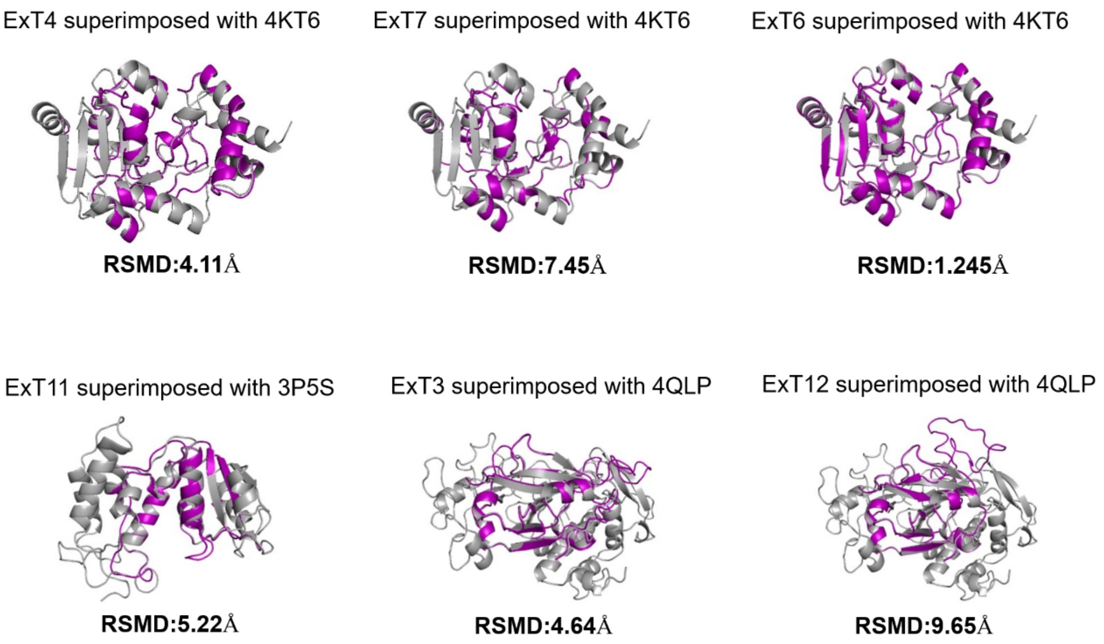

Fig. S7 Ribbon depiction showing the superimposition of AlphaFold3-predicted ExT (colored purple) and the corresponding best-matching template (colored gray) was listed here.

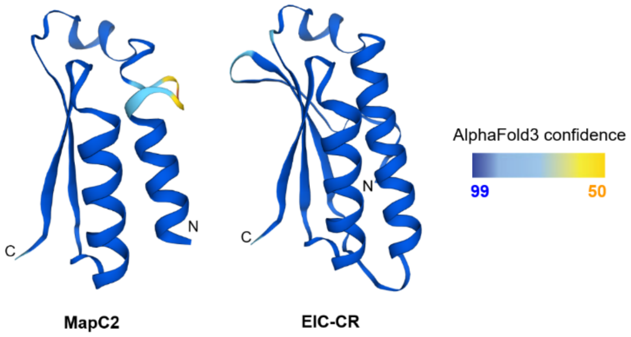

Fig. S8 AlphaFold3-predicted structures of MapC2 and EIC-CR.

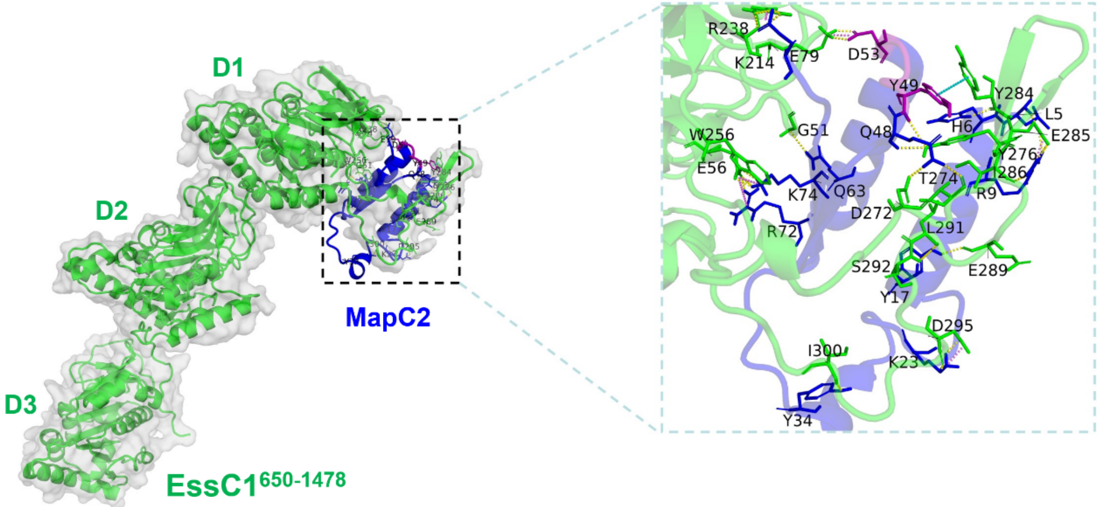

Fig. S9 Molecular docking analyses of the indicated proteins. Docked conformation and hydrogen bond interaction map of EssC1$^{650-1478}$ (shown in green) to MapC2 (shown in blue). Residues critical for ligand binding were shown as blue sticks (MapC2), and green sticks (EssC1$^{650-1478}$) highlighting hydrogen bonding interactions. The “YxxxD” motif was labelled as purple sticks.

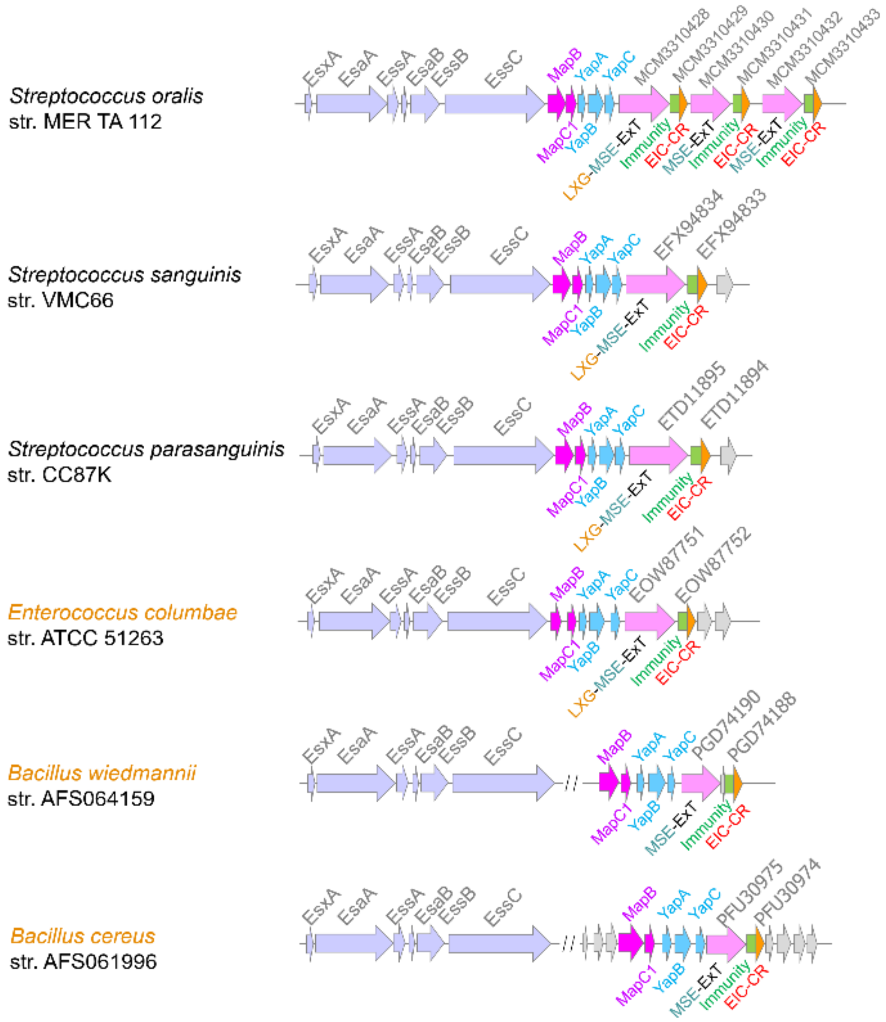

Fig. S10 Typical MSE-ExT/EIC-CR modules of T7SSb in various Gram-positive bacteria. The pink arrows indicated the putative MSE-ExT/ExT effectors, and the green plus orange arrows indicated the cognate immunity proteins with a C-terminal EIC-CR domain.

Table S1. A detailed list of bacterial strains and plasmids used in this study.

Strains or plasmids	Strains or plasmids	Source or reference
**Bacterial strains**		
K56-WJ	*Streptococcus suis* strain	Our laboratory
K11-WJ	*Streptococcus suis* strain	Our laboratory
128-2-1	*Streptococcus suis* strain	Our laboratory
SCPA	*Streptococcus suis* serotype 2 strain isolated from tonsil of healthy pig	Our laboratory
W33	*Streptococcus parasuis* strain isolated from tonsil of healthy pig	Our laboratory
U156	*Streptococcus oralis* strain isolated from tonsil of healthy pig	Our laboratory
Z5	*Staphylococcus epidermidis* strain isolated from tonsil of healthy pig	Our laboratory
E. coli Top10	For cloning the recombinant plasmids	Purchased from Vazyme
E. coli BL21(DE3)	For cloning the recombinant plasmids	Purchased from Vazyme
E. coli DH5α	For cloning the recombinant plasmids	Purchased from Vazyme
E. coli BTH101	For expressing the recombinant plasmids	Purchased from Euromedex, France
Δ*RS11280*(Δ*mse-ext1*)	Deletion mutant of *JNE31_RS11280* with *K56-WJ* background, Spc^+	This study
Δ*RS11280&01430* (Δ*mse-ext1*Δ*ei1*)	Deletion mutant of *JNE31_RS11280 and JNE31_RS01430* with *K56-WJ* background, Cat^+	This study
CΔ*RS11280*(CΔ*mse-ext1*)	Replenished of *mse-ext1* with Δ*mse-ext1* mutant background, Spc^+OptrA^+	This study
Δ*eic-CR*$^{K56\text{-}WJ}$	Deletion mutant of C-terminal of *JNE31_RS01430* with *K56-WJ* background, Spc^+	This study
Δ*mse*$^{K56\text{-}WJ}$	Deletion mutant of conserved N-terminal of *JNE31_RS11280*, Spc^+ with *K56-WJ* background	This study
Δ*mse*Δ*eic-CR*$^{K56\text{-}WJ}$	Double deletion of C-terminal of *JNE31_RS01430* with Δ*mse* background, Spc^+	This study
Δ*mapABC2*$^{K56\text{-}WJ}$	Deletion mutant of *mapABC2* with *K56-WJ* background, Spc^-/Spc^+	This study
Δ*mapC2&eic-CR*$^{K56\text{-}WJ}$	Deletion mutant of *mapC2* and eic-CR with *K56-WJ* background, Spc^+	This study
Δ*mse-ext7*	Deletion mutant of *mse-ext7* with *128-2-1* background, Spc^+	This study
Δ*mse-ext7*Δ*ei7*	Deletion mutant of *mse-ext7*, *ei7* with *128-2-1* background, Cat^+	This study
CΔ*mse-ext7*	Replenished of *mse-ext7* with Δ*mse-ext7* mutant background, Spc^+OptrA^+	This study
Δ*mse-ext9*	Deletion mutant of *mse-ext9* with *K11-WJ* background, Spc^+	This study
Δ*mse-ext9*Δ*ei9*	Deletion mutant of *mse-ext9*, *mse-ei9* with *K11-WJ* background, Cat^+	This study
CΔ*mse-ext9*	Replenished of *mse-ext9* with Δ*mse-ext9* mutant background, Spc^+OptrA^+	This study
Δ*mse-ext12*	Deletion mutant of *mse-ext12* with K11-WJ background, Spc^+	This study
Δ*mse-ext12*Δ*ei12*	Deletion mutant of *mse-ext12*, *ei12* with K11-WJ background, Cat^+	This study
CΔ*mse-ext12*	Replenished of *mse-ext12* with Δ*mse-ext12* mutant background, Spc^+OptrA^+	This study
Δ*essB&essC1*$^{K56\text{-}WJ}$	Deletion mutant of *essB, essC* with *K56-WJ* background, Spc^+	This study
Δ*esaA*$^{K56\text{-}WJ}$	Deletion mutant of *esaA* with *K56-WJ* background, Spc^-/Spc^+	This study
Δ*essC1*$^{K56\text{-}WJ}$	Deletion mutant of *essC1* with *K56-WJ* background, Spc^+	This study

$\Delta essC2^{K56\text{-}WJ}$	Deletion mutant of *essC2* with *K56-WJ* background, Spc$^+$	This study
$\Delta essC3^{K56\text{-}WJ}$	Deletion mutant of *essC3* with *K56-WJ* background, Spc$^+$	This study
$C\Delta esaA^{K56\text{-}WJ}$	Replenished of *esaA* in an ectopic region of Δ*esaA* chromosome with *K56-WJ* background, Spc$^-$/Spc$^+$	This study
$\Delta lapABC^{128\text{-}2\text{-}1}$	Deletion mutant of *lapABC* with *128-2-1* background, Spc$^-$/Spc$^+$	This study
$\Delta mapBC2^{128\text{-}2\text{-}1}$	Deletion mutant of *mapBC2* with *128-2-1* background, Spc$^-$/Spc$^+$	This study
$\Delta mapBC2\&lapABC^{128\text{-}2\text{-}1}$	Deletion mutant of *mapBC2-lapABC* with *128-2-1* background, Spc$^-$/Spc$^+$	This study
$\Delta eic\text{-}CR^{128\text{-}2\text{-}1}$	Deletion mutant of C-terminal of *ei12* with *128-2-1* background, Spc$^-$/Spc$^+$	This study
$\Delta esaA^{128\text{-}2\text{-}1}$	Deletion mutant of *esaA* with *128-2-1* background, Spc$^-$/Spc$^+$	This study
$C\Delta esaA^{128\text{-}2\text{-}1}$	Replenished of *esaA* in an ectopic region of Δ*esaA* chromosome with *128-2-1* background, Spc$^-$/Spc$^+$	This study
$\Delta mapC2^{K11\text{-}WJ}$	Deletion mutant of *mapC2* with *K11-WJ* background,Spc$^+$	This study
$C\Delta mapC2^{K11\text{-}WJ}$	Replenished of *mapC2* with Δ*mapC2* mutant background, Spc$^+$OptrA$^+$	This study
$C\Delta ei9^{K11\text{-}WJ}$	Replenished of *ei9* with Δ*eic-CR* mutant background, Spc$^+$OptrA$^+$	This study
$\Delta eic\text{-}CR^{K11\text{-}WJ}$	Deletion mutant of C-terminal of *ei9* with *K11-WJ* background, Spc$^+$	This study
**Plasmid**		
pET28a	His-tag expressing vector, Kan^R	Purchased from Vazyme
pBAD-HisA	His-tag expressing vector, Amp^R	Our laboratory
PUT18C	B2H expression vector with *plac*, Amp^R, C-terminal fusion to T18 fragment of CyaA	Purchased from Euromedex, France
PKT25	B2H expression vector with *plac*, Kan^R, C-terminal fusion to T25 fragment of CyaA	Purchased from Euromedex, France
pKSM410-tet	*S. suis / E. coli* shuttle vector for overexpression; Spc^R	Our laboratory
pSET-2S	*S. suis / E. coli* shuttle vector for complementation; Spc^R	This study
pGEX-4T-1	GST-tag expressing vector, Amp^R	Our laboratory
pET28a-mse	pET28a carrying *mse* sequence under the control of lactobiose promoter	This study
pET28a-lapC	pET28a carrying *lapC* sequence under the control of lactobiose promoter	This study
pET28a-mapC2	pET28a carrying *mapC2* sequence under the control of lactobiose promoter	This study
pET28a-D1$_{EssC1}$	pET28a carrying encoding sequence of D1 domain of EssC1 under the control of lactobiose promoter	This study
pET28a-D2$_{EssC1}$	pET28a carrying encoding sequence of D2 domain of EssC1 under the control of lactobiose promoter	This study
PUT18C-Zip	Positive control B2H expression vector	Purchased from Euromedex, France
PKT25-Zip	Positive control B2H expression vector	Purchased from Euromedex, France
pBAD::*JNE31_RS11280*$_{S403N}$	pBAD-HisA carrying *JNE31_RS11280* under the control of arabinose promoter	
pBAD::*JNE31_RS11280*$_{G478V}$	pBAD-HisA carrying JNE31_RS11280 under the control of arabinose promoter	This study
pBAD::*JNE31_RS1128&0014*	pBAD-HisA carrying *JNE31_RS11280* and *001430* under the control of arabinose	This study

30	promoter	
pBAD-ExT1&EI1-N	pBAD-HisA carrying *JNE31_RS11280* and N-terminal of *001430* under the control of arabinose promoter	This study
pBAD-ExT1&EI1-C	pBAD-HisA carrying *JNE31_RS11280* and C-terminal of *001430* under the control of arabinose promoter	This study
pBAD-ExT7	pBAD-HisA carrying *mse-ext7* under the control of arabinose promoter	This study
pBAD-ExT7&EI7	pBAD-HisA carrying *mse-ext7* and *ei7* under the control of arabinose promoter	This study
pBAD-ExT9	pBAD-HisA carrying *mse-ext9* under the control of arabinose promoter	This study
pBAD-ExT9&EI9	pBAD-HisA carrying *mse-ext9* and *ei9* under the control of arabinose promoter	This study
pBAD-ExT12	pBAD-HisA carrying *mse-ext12* under the control of arabinose promoter	This study
pBAD-ExT12&EI12	pBAD-HisA carrying *mse-ext12* and *ei12* under the control of arabinose promoter	This study
pBAD-Tse2	pBAD-HisA carrying *tse2* under the control of arabinose promoter	This study
pBAD-Tse6	pBAD-HisA carrying *tse6* under the control of arabinose promoter	This study
PKT25::EssC1-N	Kan^R, pKT25 harbouring N-terminal region of EssC1	This study
PKT25::EssC1-C	Kan^R, pKT25 harbouring C-terminal region of EssC1	This study
PKT25::EssC2	Kan^R, pKT25 harbouring EssC2 of T7SSb apparatus	This study
PKT25::EssC3	Kan^R, pKT25 harbouring EssC3 of T7SSb apparatus	This study
PUT18C::EIC-CR	Amp^R, pUT18C harbouring conserved C-terminal region of EI1	This study
PUT18C::MapC2	Amp^R, pUT18C harbouring MapC2	This study
pKSM410-*trxA-rluC*	pKSM410 carrying *trxA-rluC* under the control of tetracycline induction module	This study
pKSM410-*mse-rluC*	pKSM410 carrying *mse-rluC* under the control of tetracycline induction module	This study
pKSM410-*esxA-rluC*	pKSM410 carrying *esxA-rluC* under the control of tetracycline induction module	This study
pKSM410-*mse-ext7-rluC*	pKSM410 carrying *mse-ext7-rluC* under the control of tetracycline induction module	This study
pKSM410-*eic-CR-rluC*	pKSM410 carrying *eic-CR-rulA* under the control of tetracycline induction module	This study
pKSM410-*eic-CR-mse-rluC*	pKSM410 carrying *eic-CR-mse-rulA* under the control of tetracycline induction module	This study
pKSM410-*mapC2-mse-rluC*	pKSM410 carrying *mapC2-mse-rulA* under the control of tetracycline induction module	This study
pKSM410-*mapC1-mse-rluC*	pKSM410 carrying *mapC2-mse-rulA* under the control of tetracycline induction module	This study
pGEX-4T-*mapC2*	pGEX-4T carrying *mapC2* under the control of lactobiose promoter	This study
pGEX-4T-*eic-CR*	pGEX-4T carrying *eic-CR* under the control of lactobiose promoter	This study
pGEX-4T-*mse-C*	pGEX-4T carrying C-terminal region of *mse* under the control of lactobiose promoter	This study
pGEX-4T-*mse-N*	pGEX-4T carrying N-terminal region of *mse* under the control of lactobiose promoter	This study
pGEX-4T-$D2_{EssC4}$	pGEX-4T carrying *D2* of EssC4 under the control of lactobiose promoter	This study
pGEX-4T-$D2_{EssC1}$	pGEX-4T carrying *D2* of EssC1 under the control of lactobiose promoter	This study

Amp^R, Ampicillin resistant; Kan^R, Kanamycin resistant; Spc^R , spectinomycin resistant; Cat^R, chloramphenicol resistant

Table S2. Primers used in this study.

Primers	Primers sequence (5'-3')
**For deletion**	
Del-*mse-ext1*-1	AAGAGTTCTTACAACTGAAGAAGG
Del-*mse-ext1*-2	ATTCACGAACACTAGTCTCTTGGACTCCAACTCTTTGATT
Del-*mse-ext1*-3	ATTAAAAAAATTATAATTAGCAAATATTCGTAAGGGA
Del-*mse-ext1*-4	ATATTTCTACCCTCCCATTAT
Δ*mse-ext1*Δ*ei1*-2	AAGCTCTAGTTCGGTGCTCTTGGACTCCAACTCTTTGATT
Δ*mse-ext1*Δ*ei1*-3	ATGACTGGCTTTTATAATGTGCCAGAATAGAGTTTTAA
Del-*mse-ext7*-1	ATGGTGTTATTCAACAGTTGA
Del-*mse-ext7*-2	ATTCACGAACACTAGAAATTTGAATCTGTTCTGACA
Del-*mse-ext7*-3	ATTAAAAAAATTATAGGCAAGGTTTGTGGGAACCTG
Del-*mse-ext7*-4	ACCGCCATCTTGATCGTGATG
Δ*mse-ext7*Δ*ei7*-2	AAATTTGAATCTGTTCTGACAAAATTTGAATCTGTTCTGACA
Δ*mse-ext7*Δ*ei7*-3	ATGACTGGCTTTTATAAATGTGGAAAGTTACGGGGATG
Del-*mse-ext9*-1	AGAAAGCATATGATAATCTAC
Del-*mse-ext9*-2	ATTCACGAACACTAGTCAATCGCATCAACTCGGATTT
Del-*mse-ext9*-3	ATTCACGAACACTAGTCAATCGCATCAACTCGGATTT
Del-*mse-ext9*-4	CCTTAGAGTTCTATATCCCTA
Δ*mse-ext9*Δ*ei9*-2	AAGCTCTAGTTCGGTGCAATCGCATCAACTCGGATTT
Δ*mse-ext9*Δ*ei9*-3	ATGACTGGCTTTTATAACAGGTTCAAAAAGTTCTACAG
Del-*mse-ext12*-1	GAGTTCTTGTCAATATTTATCT
Del-*mse-ext12*-2	ATTCACGAACACTAGTTGCCAAGTGCTATTTTTGATTT
Del-*mse-ext12*-3	ATTAAAAAAATTATAAAGAAAGGAATTGTCCAATCTT
Del-*mse-ext12*-4	CGACCTTGCTGGCGATTTTAC
Δ*mse-ext12*Δ*ei12*-2	AAGCTCTAGTTCGGTGTGCCAAGTGCTATTTTTGATTT
Δ*mse-ext12*Δ*ei12*-3	ATGACTGGCTTTTATAATCCTTGTGCAAGTTCAAAAAG
Δ*mse*-1	AAGGATGCTCAGGTATCCTTG
Δ*mse*-2	TCAGCATTATCCCAACTCTTTGATTTGTTTATC
Δ*mse*-3	GGTAATCAGATTTTAAACTATAGCTTAGATGGGAAC
Δ*mse*-4	TCATCCCAAAGAGGAGAGGGATAA
Δ*mse*-C2	GCTATAGTTTAACAACTCTTTGATTTGTTTATC
Δ*mse*-C3	ATCAAAGAGTTGTTAAACTATAGCTTAGATGGGAAC
Δ*mse-5*	TTCTGTTTATTGTTTTTGGGC
Δ*mse-6*	AAGAGGAGAGGGATAATCTGAAG
Δ*ei1-c*^{CR}-1	ACAAGTTCCTGTCCGTTCATT

Δ*ei1-c*^{CR}-2	ATTCACGAACACTAGTGCTGTCCACATACTGCTTGCT
Δ*ei1-c*^{CR}-3	ATTAAAAAAATTATAAGGTTGTTGTGCCAGAATAGAG
Δ*ei1-c*^{CR}-4	GCGAATCAATAAAAATTTGAA
Δ*esaA*-1	TTGATCGTCGATGATTTCTTG
Δ*esaA*-2	ATTCACGAACACTAGTTTAATCCAATGATAGATAGAA
Δ*esaA*-3	ATTAAAAAAATTATAATACGGCGCTCGTATTGGGGCT
Δ*esaA*-4	TCTCCTTCATGTCCTCAAAGG
Δ*essB*Δ*essC*-1	CCAAAGGACTTGGCATGGGGA
Δ*essB*Δ*essC*-2	TCAGCATTATCCGTTGCCACATCAGACCGTTTT
Δ*essB*Δ*essC*-3	GGTAATCAGATT TTGTGGATAAGGTCTACAATA
Δ*essB*Δ*essC*-4	CTCCCAAGTTGGCTCGCACTC
Δ*essB*Δ*essC*-C2	CCTTATCCACAAGTTGCCACATCAGACCGTTTT
Δ*essB*Δ*essC*-C3	TGATGTGGCAACTTGTGGATAAGGTCTACAATA
Spc-F	GAAATATAATGGTTCGGGGAA
Spc-R	TTATAATTTTTTTAATCTGTTA
Cm-F	CACCGAACTAGAGCTTGATG
Cm-R	TTATAAAAGCCAGTCATTAGG
SacB+Spc-F	GGATAATGCTGAAAACTCCTT
SacB+Spc-R	AATCTGATTACCAATTAGAATGAATAT
**For complementation strains**	
T7SS-promoter-F	AAAACGACGGCCAGTGAATTCCATTTACCAACCTGCGCTCAA
T7SS-promoter-R	AGCATAATTGTAAAAATTTTATA
CΔ*mse-ext1*-F	TATAAAATTTTTACAATTATGCTTTGGAGTCCAAGAGTGATGCGGAG
CΔ*mse-ext1*-R	TTCTCTTTTCCATGGAGGATCCTTAAGCAACCTCCTTTAACAA
CΔ*mse-ext2*-F	TATAAAATTTTTACAATTATGCTGTGGACTTACAAACGGTTGAG
CΔ*mse-ext2*-R	TTCTCTTTTCCATGGAGGATCCCTATGGATTGATATACCATGA
CΔ*mse-ext3*-F	TATAAAATTTTTACAATTATGCTTTGAAACCAGACGGAACCATT
CΔ*mse-ext3*-R	TTCTCTTTTCCATGGAGGATCCGTTAGTCACCTCCCTTAA
CΔ*mse-ext7*-F	TATAAAATTTTTACAATTATGCTATGACACGTTTTTATACGAAT
CΔ*mse-ext7*-R	TTCTCTTTTCCATGGAGGATCCTTTGAATACTTCGTCTAT
CΔ*mse-ext9*-F	TATAAAATTTTTACAATTATGCTGTGGACTTACAAACGGTTGAG
CΔ*mse-ext9*-R	TTCTCTTTTCCATGGAGGATCCGTTACCTCCTAGCTGTCT
CΔ*mse-ext12*-F	TATAAAATTTTTACAATTATGCTTTGGAGTCCAAGAGTGATGCG
CΔ*mse-ext12*-R	TTCTCTTTTCCATGGAGGATCCCGGCAATATCTCCCTTAT
**For checking**	

JC-Δ*mse-ext1*-F	TGATGGCGTGGAGCAGAAAATCC
JC-Δ*mse-ext1*-R	TCCATTTTTTCATTTCTTTAGAT
JC-Δ*mse-ext7*-F	CTTGATGGCACGAAAAGTCCA
JC-Δ*mse-ext7*-R	TCTTCCAAGAGGTTCTGACGG
JC-Δ*mse-ext9*-F	CCTAGATTACTAGAAGTGTTA
JC-Δ*mse-ext9*-R	GTTTCCATCTGCCAATAATAG
JC-Δ*mse-ext12*-F	AGGATGGATTTGTACTTAGAG
JC-CΔ*mse-ext12*-R	CCATTGTCTGATGGGGCATAC
JC-CΔ*mse-ext1*-F	TGATGGCGTGGAGCAGAAAATCC
JC-CΔ*mse-ext1*-R	AATTTACATCATCACCATCACCAC
JC-CΔ*mse-ext7*-F	CTTGATGGCACGAAAAGTCCA
JC-CΔ*mse-ext7-R*	ACATCATCACCATCACCAC
JC-CΔ*mse-ext9*-F	CCTAGATTACTAGAAGTGTTA
JC-CΔ*mse-ext9-R*	TTACATCATCACCATCACCAC
JC-CΔ*mse-ext12*-F	AGGATGGATTTGTACTTAGAG
JC-CΔ*mse-ext12-R*	ATTGTCACATCATCACCATCACCAC
JC-motif-F	TTGAAGGTTGTTGTGCCAGAG
JC-motif-R	TTGGACAATTCCTTTCTTCTT
JC-pBADhis-F	AGATTAGCGGATCCTACCTG
JC-pBADhis-R	CACTTCTGAGTTCGGCATGG
JC-PKT25-F	TTCGAGTTTCGCGTGAAGGAA
JC-PKT25-R	GCTGCGCAACTGTTGGGAAGG
JC-PUT18C-F	GCATACGGCGTGGCGGGGAAA
JC-PUT18C-R	TTTCGGTGATGACGGTGAAAA
JC-PGEX-4T-1 F	GGGCTGGCAAGCCACGTTTGGTG
JC-PGEX-4T-1 R	CCGGGAGCTGCATGTGTCAGAGG
**For plasmid**	
pBAD::*JNE31_RS11280*$_{S403N}$-F	CGATGGGGATCCCTCGAGTTGGAGTCCAAGAGTGATGCGGAG
pBAD::*JNE31_RS11280*$_{S403N}$-R	CAGCCAAGCTTCGAATTCTTAAGCAACCTCCTTTAACAA
pBAD::*JNE31_RS1128&001430*-R	CAGCCAAGCTTCGAATTCCTATTCTGGCACAACAACCTT
pBAD-ExT1&EI1-N-R	CAGCCAAGCTTCGAATTCATCTTTTACGAACCAATGATT
pBAD-ExT1&EI1-C-F	ATGGAACAAGACCACCTTCTCAAA
pBAD-ExT1&EI1-C-R	CAGCCAAGCTTCGAATTCCTATTCTGGCACAACAACCTT
pBAD-ExT7-F	CGATGGGGATCCGAGCTCATGACACGTTTTTATACGAAT
pBAD-ExT7-R	CAGCCAAGCTTCGAATTCCTATTTGAATACTTCGTCTAT
pBAD-ExT7&EI7-R	CAGCCAAGCTTCGAATTCCTACTCTGGCACTACAACCTT
pBAD-ExT9-F	CGATGGGGATCCGAGCTCGTGGACTTACAAACGGTTGAGTT

pBAD-ExT9-R	CAGCCAAGCTTCGAATTCTTAGTTACCTCCTAGCTGTCTG
pBAD-ExT9&EI9-R	CAGCCAAGCTTCGAATTCCTACTCCGGCACAACAACCTT
pBAD-ExT12-F	CGATGGGGATCCGAGCTCTTGGAGTCCAAGAGTGATGCGGAGTTGGTT
pBAD-ExT12-R	CAGCCAAGCTTCGAATTCTCACGGCAATATCTCCCTTATTAG
pBAD-ExT12&EI12-R	CAGCCAAGCTTCGAATTCCTATTCTGGCACAACAACTTT
PKT25::EssC1-F	tctagaggatccccgggtaccGATGGCAGAGACAGTAGTTTTT
PKT25::EssC1-R	cttagttatatcgatgaattcGATATAAGACTGGATGACCTCTGA
PKT25::EssC2-F	tctagaggatccccgggtaccTTGCTGACAAAGGTAGGAGGC
PKT25::EssC2-R	tctagaggatccccgggtaccTCATTCATCCCCCTCCTTACT
PKT25::EssC3-F	tctagaggatccccgggtaccGATGTCTGGTGTGGTGGATGACCAGAT
PKT25::EssC3-R	cttagttatatcgatgaattcGACTCTCAGGCATGACTGGTA
PUT18C::EIC-CR-F	tctagaggatccccgggtaccGGAACAAGACCACCTTCTCAAA
PUT18C::EIC-CR-R	cttagttatatcgatgaattcTTCTGGCACAACAACCTTCAA
PUT18C::MapC2-F	tctagaggatccccgggtaccATGATACCTTACCAAGTCTGGAA
PUT18C:: MapC2-R	cttagttatatcgatgaattcCTATTCTGGCACAACAACTTT
pKSM410-*rluC*-F	caaaggagagtaatataatctcgagATGACTTCGAAAGTTTATGA
pKSM410-*rluC*-R	atcctgcaggaattcgcatgcGCTTCCTCCTCCTCCGTGGTGGTG
pKSM410-*mse-rluC*-F	gagagtaatataatctcgagTTGGAGTCCAAGAGTGATGCGGAG
pKSM410-*mse-rluC*-R	catggtctttgtagtcgctagcATCTAAGCTATAGTTTAAAGTCTTCTGC
pKSM410-*esxA-rluC*-F	gagagtaatataatctcgagCGTATTAAATTAACCCCAGAT
pKSM410-*esxA-rluC*-R	catggtctttgtagtcgctagcATTGATTTGCGCTGCAATGTC
pKSM410-*mse-ext7-rluC*-F	gagagtaatataatctcgagATGACACGTTTTTATACGAATCGCTCAGAT
pKSM410-*mse-ext7-rluC*-R	catggtctttgtagtcgctagcGAATACTTCGTCTATTGTATAATCCGACTC
pKSM410-*eic-CR-rluC*-F	gagagtaatataatctcgagATGGATACGATTGATGCAAT
pKSM410-*eic-CR-rluC*-R	catggtctttgtagtcgctagcTTCTGGCACAACAACCTTC
pKSM410-*mapC2-mse-rluC*-F	gagagtaatataatctcgagATGATACCTTACCAAGTCTGGAA
pKSM410-*mapC2-mse-rluC*-R	catggtctttgtagtcgctagcATCTAAGCTATAGTTTAAAGTCTTCTGC
pEGX-4T-D2$_{EssC1}$-F	ccgcgtggatccccggaatGGGACAGGCAAGTGGTTTGGT
pEGX-4T-D2$_{EssC1}$-R	acgatgcggccgctcgagTTGCTAGGCCCGCAAACTCTTCA
pEGX-4T-D2$_{EssC4}$-F	ccgcgtggatccccggaatGTGGGGACAGGCAAGTGGTCTGGT
pEGX-4T-D2$_{EssC4}$-R	acgatgcggccgctcgagTTCTCTGTCTCCACCATTTGTAAA
pEGX-4T- mse-N-F	ccgcgtggatccccggaatTCCTAACGGATAAACAAATCAAAGAGTTG
pEGX-4T- mse-N-R	acgatgcggccgctcgagTTGTTTACCTGCTCGCAAG
pEGX-4T- mse-C-F	ccgcgtggatccccggaatTCGTTCCGCGTGGATCCATGGCTGAGGCTGTAGTA
pEGX-4T- mse-C-R	acgatgcggccgctcgagATCTAAGCTATAGTTTAAAGTCTTCTGC
pEGX-4T- eic-CR-F	ccgcgtggatccccggaatATGGATACGATTGATGCAAT
pEGX-4T- eic-CR-R	acgatgcggccgctcgagTTCTGGCACAACAACCTTC

pEGX-4T- mapC2-F	ccgcgtggatccccggaatATGATACCTTACCAAGTCTGGAA
pEGX-4T- mapC2-R	acgatgcggccgctcgagCTATTCTGGCACAACAACTTT
pET28a-LapC-F	cagcaaatgggtcgcggatccATGTCAGAACAGATTCAAATTTCG
pET28a-LapC-R	gtggtggtggtggtgctcgagTGATCCACCTCCTGATGATGGTGC
pET28a-mapC2-F	agcaaatgggtcgcggatccATGATACCTTACCAAGTCTGGAA
pET28a-mapC2-R	gtggtggtggtggtgctcgagCTATTCTGGCACAACAACTTT
pET28a-D2$_{EssC1}$-F	cagcaaatgggtcgcggatccGGGACAGGCAAGTGGTTTGGT
pET28a-D2$_{EssC1}$-R	gtggtggtggtggtgctcgagTTGCTAGGCCCGCAAACTCTTCA
pET28a-D1$_{EssC1}$-F	cagcaaatgggtcgcggatccTTATATCTTAAGTCTTGCGGTCAA
pET28a-D1$_{EssC1}$-R	gtggtggtggtggtgctcgagTGTCCCCACAAGTCCTTGAAACCT

Table S3. The information of *Streptococcus suis* strains used in this study.

Streptococcus suis Strains	Genome accession	MSE	Source or reference
SC183	CP071305.1	+	Genbank database
CPD8	VZOB01	+	Genbank database
LSS9	JABLCW01	+	Genbank database
1378494	JABKSO01	+	Genbank database
SS1062	FIKT01	+	Genbank database
632_7C	JABLMF01	+	Genbank database
LSS8	JABLCL01	+	Genbank database
LSS92	JABLCZ01	+	Genbank database
90-4517-4	JABKWD01	+	Genbank database
632_7C	JABLMF01	+	Genbank database
LSS10	JABKZX01	+	Genbank database
LSS8	JABLCL01	+	Genbank database
SS975	JABLFT01	+	Genbank database
128-2-1	JAGFVH01	+	This study
SS341	JAILYK01	+	This study
AKJ24	JAGFQV01	+	This study
FHJ12	JAGFVD01	+	This study
FHJ20	JAGFVB01	+	This study
HCJ3	JAFFHR01	+	This study
HM55	JAEUXE01	+	This study
HN108	JAEUXF01	+	This study
HN129	JAEUXI01	+	This study
HN147	JAEUXH01	+	This study
HN0104001	JAEUXG01	+	This study
K06-WJ	JAFFHI01	+	This study
K11-WJ	JAFFHJ01	+	This study
K56-WJ	JAEUXD01	+	This study
K84-WJ	JAEVFQ01	+	This study
K328-WJ	JAFFHL01	+	This study
M70	JAGFVA01	+	This study
WH1703	JAEVFS01	+	This study
S171	JAHMRJ01	+	This study
SCMY	JAFFHE01	+	This study
SCPA	JAFFHF01	+	This study

SFJ16	JAGFUZ01	+	This study
SS286	JAGFUY01	+	This study
SS424	JAFFHO01	+	This study
ZG1004	JAGFUV01	+	This study
ZKJ33	JAFFHS01	+	This study
083_17B	JABLLB01	+	Genbank database
DN13	CP015557.1	+	Genbank database
MY1C3-3A	JABKYX01	+	Genbank database
LSS37	JABLAW01	+	Genbank database
MA4T3-1A	JABKYI01	+	Genbank database
1197165	JABKSE01	+	Genbank database
TMW_SS118	JABLKW01	+	Genbank database
VIEK01000023.1	VIEK01	+	Genbank database
90-3615-2	JABKWB01	+	Genbank database
HCB8	JAGFQW01	+	This study
2018WUSS097	JAMYJM01	-	Genbank database
2018WUSS047	JAMYJP01	-	Genbank database
2018WUSS060	JAMYJO01	-	Genbank database
2018WUSS112	JAMYJK01	-	Genbank database
2018WUSS113	JAMYJJ01	-	Genbank database
2018WUSS127	JAMYJI01	-	Genbank database
WUSS391	JAMYIS01	-	Genbank database
2018WUSS132	JAMYJH01	-	Genbank database
WUSS344	JAMYIV01	-	Genbank database
WUSS404	JAMYIR01	-	Genbank database
WUSS296	JAMYIZ01	-	Genbank database
WUSS414	JAMYIQ01	-	Genbank database
LSSP195	JAMDIS01	-	Genbank database
29896	JAASEH01	-	Genbank database
3H	JAGUAD01	-	Genbank database
SSJ28	JAIMDY01	-	Genbank database
SS986	JABLGA01	-	Genbank database
SS1062	JABLFE01	-	Genbank database
LSS58	JABLBO01	-	Genbank database
LSS48	JABLBD01	-	Genbank database
LSS39	FIGQ01	-	Genbank database

Table S4. The GenBank information and preliminary analysis of MSE-ExT effectors and immunity proteins used in this study.

Bacterial species	Putative Toxin ID	Description	LXG/YeeF	MSE	EI ID	EI-CR
Streptococcus suis	TQE86407	unknown	+	+	TQE86406	Y
Streptococcus suis	TQE86405	unknown	−	+	TQE86404	N
Streptococcus suis	TQE86401	unknown	−	+	TQE86400	Y
Streptococcus suis	TQE86399	unknown	−	+	TQE86400	Y
Streptococcus suis	TII10101	DUF4237 domain-containing protein	−	+	TII10100	Y
Streptococcus suis	TII06843	unknown	−	+	TII06844	Y
Streptococcus suis	TII04389	unknown	+	+	TII04390	Y
Streptococcus suis	TIH99923	DUF4237 domain-containing protein	−	+	TIH99922	Y
Streptococcus suis	TIH99493	DUF4237 domain-containing protein	−	+	TIH99443	Y
Streptococcus suis	QZT18553	unknown	−	+	QZT18554	Y
Streptococcus suis	QSQ90801	unknown	+	+	QSQ90800	Y
Streptococcus suis	NRH15204	unknown	−	+	NRH15205	N
Streptococcus suis	NRH15201	glycohydrolase toxin TNT-related protein	−	+	NRH15202	Y
Streptococcus suis	NRH07348	glycohydrolase toxin TNT-related protein	−	+	NRH07349	Y
Streptococcus suis	NRG70216	glycohydrolase toxin TNT-related protein	−	+	NRG70217	Y
Streptococcus suis	NRG60352	glycohydrolase toxin TNT-related protein	−	+	NRG60353	Y
Streptococcus suis	NQS07155	unknown	+	+	HO772_10850	Y
Streptococcus suis	NQS07122	unknown	−	+	NQS07121	N
Streptococcus suis	NQR92086	glycohydrolase toxin TNT-related protein	−	+	NQR92087	Y
Streptococcus suis	NQQ50749	unknown	+	+	NQQ50750	Y
Streptococcus suis	NQQ04556	unknown	+	+	NQQ04557	Y
Streptococcus suis	NQP82438	unknown	+	+	NQP82437	Y
Streptococcus suis	NQP80385	unknown	+	+	NQP80384	Y

Streptococcus suis	NQP78328	unknown	+	+	NQP78327	Y
Streptococcus suis	NQP72025	unknown	+	+	NQP72026	Y
Streptococcus suis	NQP70211	unknown	+	+	NQP70210	Y
Streptococcus suis	NQP67692	glycohydrolase toxin TNT-related protein	−	+	NQP67693	Y
Streptococcus suis	NQP61885	unknown	+	+	HO910_09770	Y
Streptococcus suis	NQP61198	unknown	−	+	NQP61199	N
Streptococcus suis	NQP57495	unknown	−	+	NQP57496	N
Streptococcus suis	NQP49346	unknown	−	+	NQP49347	Y
Streptococcus suis	NQP43649	glycohydrolase toxin TNT-related protein	−	+	NQP43650	Y
Streptococcus suis	NQP38679	glycohydrolase toxin TNT-related protein	−	+	NQP38680	Y
Streptococcus suis	NQP28682	glycohydrolase toxin TNT-related protein	−	+	NQP28683	Y
Streptococcus suis	NQP27685	glycohydrolase toxin TNT-related protein	−	+	NQP27686	Y
Streptococcus suis	NQP01398	unknown	−	+	NQP01399	Y
Streptococcus suis	NQP01397	unknown	+	+	HO940_07605	Y
Streptococcus suis	NQO89286	glycohydrolase toxin TNT-related protein	−	+	NQO89287	Y
Streptococcus suis	NQO80686	glycohydrolase toxin TNT-related protein	−	+	NQO80687	Y
Streptococcus suis	NQN60631	unknown	−	+	HPA08_10335	Y
Streptococcus suis	NQM88932	glycohydrolase toxin TNT-related protein	−	+	NQM88933	Y
Streptococcus suis	NQM46524	TNT domain-containing protein	−	+	HO408_02610	Y
Streptococcus suis	NQM46523	TNT domain-containing protein	−	+	HO408_02595	Y
Streptococcus suis	NQM26257	unknown	−	+	NQM26258	Y
Streptococcus suis	NQM26255	unknown	+	+	NQM26256	Y
Streptococcus suis	NQM21410	glycohydrolase toxin TNT-related protein	−	+	NQM21411	Y
Streptococcus suis	NQM19323	glycohydrolase toxin TNT-related	−	+	NQM19324	Y

		protein				
Streptococcus suis	NQM19321	unknown	+	+	NQM19322	Y
Streptococcus suis	NQM18236	glycohydrolase toxin TNT-related protein	−	+	NQM18237	Y
Streptococcus suis	NQM09920	glycohydrolase toxin TNT-related protein	−	+	NQM09921	Y
Streptococcus suis	NQM09918	unknown	−	−	NQM09919	Y
Streptococcus suis	NQL91944	unknown	−	+	NQL91943	Y
Streptococcus suis	NQL91940	unknown	+	+	NQL91941	Y
Streptococcus suis	NQL57455	unknown	−	+	NQL57455	Y
Streptococcus suis	NQL57452	glycohydrolase toxin TNT-related protein	−	+	NQL57453	Y
Streptococcus suis	NQL57450	unknown	−	+	NQL57451	Y
Streptococcus suis	NQK46942	unknown	−	+	NQK46941	Y
Streptococcus suis	NQK36110	unknown	+	+	NQK36111	Y
Streptococcus suis	NQK34212	unknown	+	+	NQK34211	Y
Streptococcus suis	NQK17588	unknown	+	+	NQK17589	Y
Streptococcus suis	NQK17490	glycohydrolase toxin TNT-related protein	−	+	NQK17491	Y
Streptococcus suis	NQK12276	glycohydrolase toxin TNT-related protein	−	+	NQK12275	Y
Streptococcus suis	NQK12274	unknown	−	+	NQK12273	N
Streptococcus suis	NQJ97945	unknown	−	+	NQJ97946	Y
Streptococcus suis	NQJ90049	glycohydrolase toxin TNT-related protein	−	+	NQJ90048	Y
Streptococcus suis	NQJ87306	glycohydrolase toxin TNT-related protein	−	+	NQJ87307	Y
Streptococcus suis	NQJ87304	unknown	−	+	NQJ87305	Y
Streptococcus suis	NQJ87302	unknown	+	+	NQJ87303	Y
Streptococcus suis	NQJ67494	TNT domain-containing protein	−	+	NQJ67495	Y
Streptococcus suis	NQJ63776	glycohydrolase toxin TNT-related protein	−	+	NQJ63777	Y

Streptococcus suis	NQJ63774	unknown	−	+	NQJ63775	Y
Streptococcus suis	NQJ63772	unknown	+	+	NQJ63773	Y
Streptococcus suis	NQJ59997	glycohydrolase toxin TNT-related protein	−	+	NQJ59998	Y
Streptococcus suis	NQJ59995	unknown	−	+	NQJ59996	Y
Streptococcus suis	NQJ59993	unknown	+	+	NQJ59994	Y
Streptococcus suis	NQJ55689	glycohydrolase toxin TNT-related protein	−	+	NQJ55688	Y
Streptococcus suis	NQJ51511	glycohydrolase toxin TNT-related protein	−	+	NQJ51512	Y
Streptococcus suis	NQJ49357	glycohydrolase toxin TNT-related protein	−	+	NQJ49358	Y
Streptococcus suis	NQJ43153	glycohydrolase toxin TNT-related protein	−	+	NQJ43154	Y
Streptococcus suis	NQJ02256	glycohydrolase toxin TNT-related protein	−	+	NQJ02257	Y
Streptococcus suis	NQJ02254	unknown	+	+	NQJ02255	Y
Streptococcus suis	NQI94090	unknown	−	+	NQI94089	Y
Streptococcus suis	NQI94088	glycohydrolase toxin TNT-related protein	−	+	NQI94087	Y
Streptococcus suis	NQI92219	unknown	+	+	NQI92220	N
Streptococcus suis	NQI89638	unknown	+	+	NQI89639	Y
Streptococcus suis	NQI89636	glycohydrolase toxin TNT-related protein	−	+	NQI89637	Y
Streptococcus suis	NQI89634	unknown	−	+	NQI89635	Y
Streptococcus suis	NQI71380	unknown	+	+	HO594_09555	Y
Streptococcus suis	NQI71004	unknown	−	+	NQI71003	Y
Streptococcus suis	NQI18270	unknown	−	+	NQI18271	N
Streptococcus suis	NQI18269	unknown	+	+	HO620_06515	Y
Streptococcus suis	NQI06241	unknown	+	+	NQI06240	Y
Streptococcus suis	NQI06239	glycohydrolase toxin TNT-related protein	−	+	NQI06238	Y
Streptococcus suis	NQH68723	unknown	+	+	NQH68720	Y

Streptococcus suis	NQH68721	glycohydrolase toxin TNT-related protein	−	+	NQH68722	Y
Streptococcus suis	NQH64870	unknown	+	+	HO645_10590	Y
Streptococcus suis	NQH64869	unknown	−	+	NQH64868	N
Streptococcus suis	NQH52958	glycohydrolase toxin TNT-related protein	−	+	NQH52957	Y
Streptococcus suis	NQH48075	glycohydrolase toxin TNT-related protein	−	+	NQH48074	Y
Streptococcus suis	NQH32488	unknown	−	+	NQH32487	N
Streptococcus suis	NQH28402	glycohydrolase toxin TNT-related protein	−	+	NQH28401	Y
Streptococcus suis	NQG74626	unknown	−	+	NQG74627	Y
Streptococcus suis	NQG74592	unknown	+	−	NQG74593	Y
Streptococcus suis	NQG46949	glycohydrolase toxin TNT-related protein	−	+	NQG46948	Y
Streptococcus suis	NQG44015	unknown	+	+	NQG44016	Y
Streptococcus suis	NQG44013	unknown	−	−	NQG44014	Y
Streptococcus suis	NQG37101	unknown	+	−	NQG37102	Y
Streptococcus suis	NJW40286	TNT domain-containing protein	−	+	NJW40285	Y
Streptococcus suis	NJW38487	unknown	−	+	HC239_03445	N
Streptococcus suis	MCO8241656	glycohydrolase toxin TNT-related protein	−	+	MCO8241657	Y
Streptococcus suis	MCO8239204	unknown	−	+	MCO8239205	Y
Streptococcus suis	MCO8239206	unknown	−	+	MCO8239207	Y
Streptococcus suis	MCO8230896	unknown	−	+	MCO8230897	Y
Streptococcus suis	MCO8230894	unknown	−	+	MCO8230895	Y
Streptococcus suis	MCO8228217	glycohydrolase toxin TNT-related protein	−	+	MCO8228218	Y
Streptococcus suis	MCO8228219	unknown	−	+	MCO8228220	Y
Streptococcus suis	MCO8226222	glycohydrolase toxin TNT-related protein	−	+	MCO8226223	Y
Streptococcus suis	MCO8226224	unknown	−	+	MCO8226225	Y

Streptococcus suis	MCO8221848	glycohydrolase toxin TNT-related protein	−	+	MCO8221847	Y
Streptococcus suis	MCO8221846	unknown	−	+	MCO8221845	Y
Streptococcus suis	MCO8220132	glycohydrolase toxin TNT-related protein	−	+	MCO8220131	Y
Streptococcus suis	MCO8220130	unknown	−	+	MCO8220129	N
Streptococcus suis	MCO8217486	unknown	+	+	MCO8217487	Y
Streptococcus suis	MCO8217483	unknown	−	+	MCO8217484	Y
Streptococcus suis	MCO8217482	unknown	−	+	MCO8217481	Y
Streptococcus suis	MCO8213509	unknown	−	+	MCO8213508	Y
Streptococcus suis	MCO8213507	unknown	−	+	MCO8213506	Y
Streptococcus suis	MCO8207246	unknown	−	+	MCO8207245	Y
Streptococcus suis	MCO8201753	glycohydrolase toxin TNT-related protein	−	+	MCO8201752	Y
Streptococcus suis	MCO8201751	unknown	−	+	MCO8201750	Y
Streptococcus suis	MCO8199851	unknown	−	+	MCO8199852	Y
Streptococcus suis	MCO8199850	unknown	−	+	MCO8199849	Y
Streptococcus suis	MCO8199847	unknown	+	+	MCO8199846	Y
Streptococcus suis	MCO8190976	unknown	−	+	MCO8190977	Y
Streptococcus suis	MCO8190974	glycohydrolase toxin TNT-related protein	−	+	MCO8190975	Y
Streptococcus suis	MCO8189241	unknown	−	+	MCO8189240	Y
Streptococcus suis	MCO8189239	unknown	−	+	MCO8189238	Y
Streptococcus suis	MCO8183827	unknown	−	+	MCO8183828	Y
Streptococcus suis	MCO8180844	unknown	−	+	MCO8180845	Y
Streptococcus suis	MCO8180842	glycohydrolase toxin TNT-related protein	−	+	MCO8180843	Y
Streptococcus suis	MCO8179594	unknown	−	+	MCO8179595	N
Streptococcus suis	MCO8179593	unknown	−	−	MCO8179592	Y
Streptococcus suis	MCO8175461	unknown	−	+	MCO8175462	Y
Streptococcus suis	MCO8172459	glycohydrolase toxin TNT-related	−	+	MCO8172460	Y

		protein				
Streptococcus suis	MCL4960519	unknown	+	+	MCL4960518	Y
Streptococcus suis	MCL4956223	unknown	+	+	MCL4956222	Y
Streptococcus suis	MCL4928804	unknown	+	+	MCL4928803	Y
Streptococcus suis	MCL4928802	glycohydrolase toxin TNT-related protein	−	+	MCL4928801	Y
Streptococcus suis	MCL4922525	unknown	−	+	MCL4922526	Y
Streptococcus suis	MCL4908360	unknown	+	+	MCL4908359	Y
Streptococcus suis	MCL4908358	glycohydrolase toxin TNT-related protein	−	+	MCL4908357	Y
Streptococcus suis	MCL4908356	unknown	−	+	MCL4908355	N
Streptococcus suis	MCL4882713	unknown	−	−	MCL4882714	Y
Streptococcus suis	MCL4882711	unknown	−	truncated	MCL4882712	Y
Streptococcus suis	MCL4880812	unknown	−	+	MCL4880813	Y
Streptococcus suis	MCK4028930	unknown	−	+	MCK4028929	Y
Streptococcus suis	MCK4028928	membrane protein	−	+	MCK4028927	Y
Streptococcus suis	MCK4027716	unknown	−	+	MCK4027717	Y
Streptococcus suis	MCK4025490	unknown	−	truncated	MCK4025489	Y
Streptococcus suis	MCK4018897	TNT domain-containing protein	−	+	HCC62_03485	Y
Streptococcus suis	MCK4018894	unknown	+	−	MCK4018896	Y
Streptococcus suis	MCK3990349	TNT domain-containing protein	−	+	MCK3990348	Y
Streptococcus suis	MCK3987849	unknown	+	−	MCK3987847	Y
Streptococcus suis	MCK3987848	unknown	−	+	MCK3987847	Y
Streptococcus suis	MCK3947500	TNT domain-containing protein	−	+	MCK3947499	Y
Streptococcus suis	MCK3943328	unknown	−	+	MCK3943327	Y
Streptococcus suis	MCK3922631	TNT domain-containing protein	−	+	MCK3922630	Y
Streptococcus suis	MCK3922629	TNT domain-containing protein	−	+	MCK3922628	Y
Streptococcus suis	MCK3922627	TNT domain-containing protein	−	+	MCK3922626	Y
Streptococcus suis	MCK3907530	TNT domain-containing protein	−	+	MCK3907531	Y

Streptococcus suis	MCK3907528	unknown	−	+	MCK3907529	Y
Streptococcus suis	MCK3907526	unknown	+	+	MCK3907527	Y
Streptococcus suis	MCH1644476	unknown	+	+	MCH1644475	Y
Streptococcus suis	MCH1644474	glycohydrolase toxin TNT-related protein	−	+	MCH1644473	Y
Streptococcus suis	MCH1644472	unknown	−	+	MCH1644471	N
Streptococcus suis	MBY5025457	unknown	−	+	MBY5025458	Y
Streptococcus suis	MBY4634610	unknown	−	+	MBY4634611	Y
Streptococcus suis	MBS8117002	unknown	+	+	MBS8117001	Y
Streptococcus suis	MBS8114009	TNT domain-containing protein	+	+	MBS8114010	Y
Streptococcus suis	MBS8114007	unknown	−	+	MBS8114008	Y
Streptococcus suis	MBS8108145	unknown	+	+	MBS8108146	Y
Streptococcus suis	MBS8104704	unknown	+	+	MBS8104705	Y
Streptococcus suis	MBS8099553	unknown	+	+	MBS8099554	Y
Streptococcus suis	MBS8095893	unknown	+	+	MBS8095894	Y
Streptococcus suis	MBS8067869	unknown	+	+	MBS8067870	Y
Streptococcus suis	MBS8058641	unknown	+	+	MBS8058640	Y
Streptococcus suis	MBS8058639	glycohydrolase toxin TNT-related protein	−	+	MBS8058638	Y
Streptococcus suis	MBS8058637	unknown	−	+	MBS8058636	N
Streptococcus suis	MBS8051235	unknown	+	+	MBS8051236	Y
Streptococcus suis	MBS8039075	unknown	+	+	MBS8039074	Y
Streptococcus suis	MBS0714024	unknown	−	+	MBS0714025	Y
Streptococcus suis	MBS0714020	unknown	−	+	MBS0714023	Y
Streptococcus suis	MBS0714019	unknown	−	−	MBS0714018	Y
Streptococcus suis	MBS0714015	unknown	−	+	MBS0714014	Y
Streptococcus suis	MBS0705832	unknown	+	+	MBS0705833	Y
Streptococcus suis	MBS0687229	unknown	−	+	MBS0687230	Y
Streptococcus suis	MBS0687225	unknown	−	+	MBS0687228	Y

Streptococcus suis	MBS0687224	unknown	−	−	MBS0687223	Y
Streptococcus suis	MBS0687220	unknown	−	+	MBS0687219	Y
Streptococcus suis	MBO4137785	unknown	+	+	MBO4137786	Y
Streptococcus suis	MBO4127970	glycohydrolase toxin TNT-related protein	−	+	MBO4127969	Y
Streptococcus suis	MBO4127968	unknown	−	+	MBO4127969	Y
Streptococcus suis	MBO4126686	glycohydrolase toxin TNT-related protein	−	+	MBO4126687	Y
Streptococcus suis	MBO4118013	unknown	−	+	MBO4118012	Y
Streptococcus suis	MBO4114919	glycohydrolase toxin TNT-related protein	−	+	MBO4114920	Y
Streptococcus suis	MBO4113855	unknown	−	+	MBO4113856	Y
Streptococcus suis	MBO4113852	glycohydrolase toxin TNT-related protein	−	+	J5652_04590	Y
Streptococcus suis	J5652_04620	unknown	−	+	MBO4113857	Y
Streptococcus suis	MBO4113849	unknown	−	+	MBO4113850	Y
Streptococcus suis	MBO4113848	unknown	+	+	J5652_04560	Y
Streptococcus suis	MBO4111502	unknown	−	+	MBO4111501	Y
Streptococcus suis	MBO4110115	unknown	+	+	J5583_08095	Y
Streptococcus suis	MBO3838242	TNT domain-containing protein	−	truncated	J5O01_05295	Y
Streptococcus suis	MBO3838240	unknown	+	+	MBO3838239	Y
Streptococcus suis	MBO3838237	glycohydrolase toxin TNT-related protein	−	+	MBO3838236	Y
Streptococcus suis	MBO3838235	unknown	−	+	MBO3838236	Y
Streptococcus suis	MBM7320808	unknown	−	−	MBM7320809	Y
Streptococcus suis	MBM7318570	glycohydrolase toxin TNT-related protein	−	+	MBM7318571	Y
Streptococcus suis	MBM7311972	glycohydrolase toxin TNT-related protein	−	+	MBM7311973	Y
Streptococcus suis	MBM7285387	unknown	+	+	MBM7285386	Y
Streptococcus suis	MBM7285385	unknown	−	+	MBM7285386	Y
Streptococcus suis	MBM7283219	unknown	−	+	MBM7283218	Y

Streptococcus suis	MBM7281816	unknown	−	+	MBM7281817	Y
Streptococcus suis	MBM7270195	glycohydrolase toxin TNT-related protein	−	+	MBM7270196	Y
Streptococcus suis	MBM7178861	unknown	+	−	MBM7178859	Y
Streptococcus suis	MBM7178860	unknown	−	+	MBM7178859	Y
Streptococcus suis	MBM0272512	glycohydrolase toxin TNT-related protein	−	+	MBM0272513	Y
Streptococcus suis	MBM0241737	unknown	−	+	MBM0241736	Y
Streptococcus suis	MBL6583748	unknown	+	+	MBL6583747	Y
Streptococcus suis	MBL6583746	unknown	−	+	MBL6583747	Y
Streptococcus suis	MBL6562581	unknown	+	+	MBL6562580	Y
Streptococcus suis	MBL6562579	unknown	−	+	MBL6562580.	Y
Streptococcus suis	MBL6538044	glycohydrolase toxin TNT-related protein	−	+	MBL6538045	Y
Streptococcus suis	MBL6516097	unknown	−	truncated	MBL6516096	Y
Streptococcus suis	MBL6515359	unknown	−	+	MBL6515358	Y
Streptococcus suis	MBL6515357	glycohydrolase toxin TNT-related protein	−	+	MBL6515358	Y
Streptococcus suis	MBL6503047	unknown	−	+	MBL6503048	Y
Streptococcus suis	MBL6440282	unknown	−	+	MBL6440283	Y
Streptococcus suis	MBL6440278	unknown	−	+	MBL6440277	Y
Streptococcus suis	MBL6440276	glycohydrolase toxin TNT-related protein	−	+	MBL6440277	Y
Streptococcus suis	KPA71558	unknown	−	+	KPA71557	Y
Streptococcus suis	KPA59759	unknown	−	truncated	KPA59774	Y
Streptococcus suis	CYX87311	unknown	−	+	CYX87264	N
Streptococcus suis	CYX87364	unknown	+	+	CYX87339	Y
Streptococcus suis	CYX38875	unknown	−	+	CYX38907	Y
Streptococcus suis	CYX34420	unknown	−	+	CYX34439	N
Streptococcus suis	CYX18263	unknown	+	+	CYX18243	Y
Streptococcus suis	CYX02397	membrane protein	+	+	CYX02418	Y

Streptococcus suis	CYW29205	unknown	+	−	CYW29235	Y
Streptococcus suis	CYW22848	membrane protein	−	+	CYW22870	Y
Streptococcus suis	CYW20135	unknown	+	+	CYW20431	Y
Streptococcus suis	CYV55587	membrane protein	+	+	CYV55636	Y
Streptococcus suis	CYV51053	unknown	+	+	CYV51066	Y
Streptococcus suis	CYV49462	glycohydrolase toxin TNT-related protein	+	+	CYV49442	Y
Streptococcus suis	CYV49397	unknown	−	−	CYV49442	Y
Streptococcus suis	CYV28904	membrane protein	−	+	CYV28888	Y
Streptococcus suis	CYV24530	unknown	−	+	CYV24519	Y
Streptococcus suis	CYV24507	unknown	−	+	CYV24492	N
Streptococcus suis	CYV21124	membrane protein	−	+	CYV21132	Y
Streptococcus suis	CYV17487	membrane protein	−	+	CYV17466	Y
Streptococcus suis	CYV08178	membrane protein	−	+	CYV08170	Y
Streptococcus suis	CYU57982	unknown	+	+	CYU57896	Y
Streptococcus suis	CYU57918	unknown	−	+	CYU57954	Y
Streptococcus suis	CYU39375	unknown	−	+	CYU39351	Y
Streptococcus suis	CYT87808	unknown	−	+	CYT87845	Y
Streptococcus suis	CYT87755	unknown	+	+	CYT87783	Y
Streptococcus suis	CYT85633	unknown	+	+	CYT85592	Y
Streptococcus suis	BCK46445	unknown	+	+	BCK46444	Y
Streptococcus suis	BCK46443	glycohydrolase toxin TNT-related protein	−	+	BCK46442	Y
Streptococcus suis	BCK46441	unknown	−	+	BCK46440	N
Streptococcus suis	AUW27032	unknown	−	+	AUW27031	Y
Streptococcus suis	AUW27030	unknown	+	+	AUW27031	Y
Streptococcus suis	ASW50392	TNT domain-containing protein	+	+	ASW50391	Y
Streptococcus suis	AOM75424	unknown	+	+	AOM75423	Y
Streptococcus suis	AND00702	unknown	+	+	AND00701	Y

Streptococcus suis	AND00700	unknown	−	+	AND00701	Y
Streptococcus suis	AKH11137	unknown	+	−	AHF60307	Y
Streptococcus suis	AKH11136	Hemagglutinin/hemolysin-like protein	−	+	AHF60307	Y
Streptococcus suis	AHF60306	unknown	−	+	AHF60307	Y
Streptococcus suis	AGW88186	unknown	+	+	AGW88185	Y
Streptococcus suis	AGW88184	unknown	−	+	AGW88183	N
Streptococcus suis	AEB82247	unknown	+	+	AEB82245	Y
Streptococcus suis	NQL91942	glycohydrolase toxin TNT-related protein	−	+	NQL91943	Y
Streptococcus suis	NQJ14968	glycohydrolase toxin TNT-related protein	−	+	NQJ14967	Y
Streptococcus suis	MCO8213509	unknown	−	+	MCO8213510	Y
Streptococcus suis	MCO8205467	unknown	−	+	MCO8205468	Y
Streptococcus suis	MCO8172461	unknown	−	+	MCO8172462	Y
Streptococcus suis	MCL4882715	unknown	−	truncated	MCL4882714	Y
Streptococcus suis	MBY4964243	glycohydrolase toxin TNT-related protein	−	+	MBY4964244	Y
Streptococcus suis	MBS0714024	unknown	−	+	MBS0714025	Y
Streptococcus suis	MBS0714021	unknown	−	+	MBS0714023	Y
Streptococcus suis	MBS0687226	unknown	−	+	MBS0687228	Y
Streptococcus suis	MBO3755740	unknown	−	+	MBO3838239	Y
Streptococcus suis	MBM7320810	unknown	+	+	MBM7320809	Y
Streptococcus suis	MBM7203804	unknown	−	+	MBM7203803	Y
Streptococcus suis	MBM7178858	unknown	−	+	MBM7178859	Y
Streptococcus suis	MBL6440284	unknown	−	+	MBL6440285	Y
Streptococcus suis	CYX87364	unknown	+	+	CYX87339	Y
Streptococcus suis	CYW27391	unknown	−	−	CYW27412	Y
Streptococcus suis	CYU93534	unknown	−	+	CYU93549	Y
Streptococcus suis	CYU39329	unknown	+	+	CYU39351	Y
Streptococcus suis	CYT87808	unknown	−	+	CYT87845	Y

Streptococcus suis	AOM75421	unknown	–	+	AOM75422	Y
Streptococcus suis	MBO3838238	unknown	–	–	MBO3838239	Y
streptococcus suis	CYV08231	unknown	–	–	CYV08206	Y
streptococcus suis	CYV17552	unknown	–	–	CYV17527	Y
streptococcus suis	CYV28934	unknown	–	–	CYV28917	Y
streptococcus suis	CYX34457	unknown	–	–	CYX34477	Y
streptococcus suis	CYX38837	unknown	–	–	CYX38857	Y
streptococcus suis	MBS0687229	unknown	–	–	MBS0687230	Y
streptococcus suis	MBY4964241	unknown	–	–	MBY4964242	Y
streptococcus suis	MCK4027714	unknown	–	–	MCK4027715	Y
streptococcus suis	MCL4922523	unknown	–	–	MCL4922524	Y
streptococcus suis	MCO8172457	unknown	–	–	MCO8172458	Y
streptococcus suis	MCO8189243	unknown	–	–	MCO8189242	Y
streptococcus suis	MCO8190972	unknown	–	–	MCO8190973	Y
streptococcus suis	MCO8201755	unknown	–	–	MCO8201754	Y
streptococcus suis	MCO8213511	unknown	–	–	MCO8213510	Y
streptococcus suis	MCO8220134	unknown	–	–	MCO8220133	Y
streptococcus suis	MCO8221850	unknown	–	–	MCO8221849	Y
streptococcus suis	MCO8226220	unknown	–	–	MCO8226221	Y
streptococcus suis	MCO8228215	unknown	–	–	MCO8228216	Y
streptococcus suis	MCO8230892	unknown	–	–	MCO8230893	Y
streptococcus suis	MCO8239202	unknown	–	–	MCO8239203	Y
streptococcus suis	MCO8241654	unknown	–	–	MCO8241655	Y
